# Screening for Selective Anticancer Activity of Extracts from 59 Plant Species Collected in Southern Spain (Andalusia)

**DOI:** 10.3390/ph19040616

**Published:** 2026-04-14

**Authors:** Víctor Jiménez-González, Guillermo Benítez, Julio Enrique Pastor, Miguel López-Lázaro, José Manuel Calderón-Montaño

**Affiliations:** 1 Department of Pharmacology, Faculty of Pharmacy, University of Seville, 41012 Seville, Spain; mlopezlazaro@us.es; 2 Department of Botany, Faculty of Pharmacy, University of Granada, 18071 Granada, Spain; gbcruz@ugr.es; 3 Department of Vegetal Biology and Ecology, Faculty of Biology, University of Seville, 41012 Seville, Spain; jpastor@us.es

**Keywords:** cancer, lung cancer, selectivity, *Thymelaea*, *Daphne*

## Abstract

**Background:** Despite pharmacological advances, many cancer therapies provide only limited clinical benefits while often inducing significant toxicity. Therefore, the search for more effective and safer anticancer drugs remains an urgent priority. This study aimed to identify plant extracts from the Andalusian flora (Southern Spain) with selective anticancer potential. **Methodology:** A total of 67 extracts from 59 plant species were screened for selective cytotoxicity using A549 lung adenocarcinoma and HaCaT non-malignant cells. The most promising candidates, extracts from *Thymelaea lanuginosa* and *Daphne oleoides*, were further evaluated through fluorescence-based co-cultures, cell cycle analysis, and redox-mechanism assay. These extracts were also tested against a panel of cancer cells derived from different tissues (MDA-MB-231, T24, KATO-III, SK-OV-3, and MeWo). **Results:** Several extracts exhibited selective activity against A549 cancer cells, including extracts from *Chamaeiris foetidissima* (L.) Medik. (=*Iris foetidissima* L.), *Daphne oleoides* Schreb, *Iberodes linifolia* (L.) M. Serrano, R. Carbajal & S. Ortiz, *Reseda media* Lag., *Saxifraga hirsuta* L., *Seseli montanum* subsp. *granatense* (Willk.) C. Pardo, *Thymelaea lanuginosa* (Lam.), and *Tordylium officinale* L. The extracts from *D. oleoides* and *T. lanuginosa* were over 1000 times more active against lung cancer cells than non-malignant cells. These extracts induced a specific G1-phase arrest in A549 cells. Both extracts showed also selective activity against triple-negative breast cancer cells (MDA-MB-231) and bladder cancer cells (T24). **Conclusions:** These findings highlight *Daphne* and *Thymelaea* species as valuable sources for discovering novel selective anticancer agents. Future research should focus on bio-guided fractionation and in vivo validation to fully delineate their therapeutic potential.

## 1. Introduction

Globally, one in four deaths resulting from non-communicable illnesses is caused by cancer [1,2]. Improvements in diagnostic techniques, widespread early detection campaigns, and advancements in treatment have led to an increase in cancer survival rates. The 5-year survival rate after diagnosis has increased from an average of 49% in the 1970s to 69% during the 2014–2020 period. Early detection of cancer has been a major contributor to improved survival rates. At this stage, survival is high because tumors remain localized and can often be eliminated by surgery or radiotherapy. However, once cancer has spread to distant sites (metastasis), the main therapeutic approach relies on systemic drugs to reach disseminated cancer cells. Most of these drugs target highly proliferative cells, thereby affecting not only cancer cells but also healthy cells such as immune and epithelial cells. As a result, they frequently cause adverse effects including neutropenia, diarrhea, and severe fatigue. To minimize toxicity, such drugs must be administered at doses tolerable for patients, which are often lower than those required for complete eradication of cancer cells. To overcome these limitations, targeted therapies and immunotherapies have been developed in recent decades, aiming for greater selectivity against cancer. Targeted therapies act on specific proteins essential for tumor growth and survival, while immunotherapies activate the immune system to attack cancer cells. These strategies have improved survival in certain cancers, for example with the combination of tyrosine kinase inhibitors and immune checkpoint inhibitors in renal cancer [3]. Nevertheless, the proportion of patients diagnosed with metastatic disease who survive beyond five years remains very low [1,2]. For instance, survival rates for patients with liver, lung, esophageal, pancreatic, and urinary bladder cancers remain below 10% at the metastatic stage. These data highlight the limitations of current treatments. Indeed, a recent study reported that despite substantial economic investment in anticancer drug development, only a small fraction of drugs approved between 1995 and 2020 have provided meaningful clinical benefit for patients [4]. Therefore, the identification of novel anticancer agents capable of selectively targeting malignant cells is essential to achieve more effective and cost-efficient therapies.

**Figure 1:**
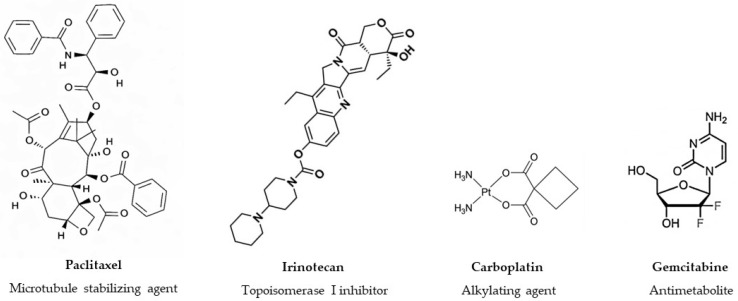
Chemical structures and classification of representative anticancer agents: Paclitaxel (a taxane diterpenoid isolated from *Taxus* species), irinotecan (a semi-synthetic derivative of camptothecin that acts as a topoisomerase I inhibitor), carboplatin (a second-generation inorganic platinum-based alkylating agent) and gemcitabine (a nucleoside analogue that functions as an antimetabolite).

Nature has played a pivotal role in anticancer drug discovery, providing numerous clinically approved agents, either as unmodified compounds or as the basis for semisynthetic derivatives [5,6,7]. Notable examples of botanical oncology drugs include paclitaxel (extracted from *Taxus brevifolia* L.) and its derivative docetaxel. Paclitaxel is widely indicated for non-small cell lung cancer (NSCLC), Kaposi sarcoma, ovarian and breast cancers. Similarly, its semi-synthetic analogue, docetaxel, has become a cornerstone for treating prostate and mammary carcinomas. Etoposide (a semisynthetic compound derived from *Podophyllum peltatum* L.) is another plant-derived drug used for Hodgkin lymphoma, NSCLC, retinoblastoma, and Ewing sarcoma. Irinotecan (a derivative of camptothecin isolated from *Camptotheca acuminata* Decne.) is used for metastatic colorectal cancer. All these plant-derived drugs are included in the ‘WHO model list of essential medicines’ (23rd edition) [8] and continue to be investigated in combination with other agents in several clinical trials. For example; the combination of paclitaxel with pembrolizumab and carboplatin is under investigation for NSCLC patients in a Phase III trial at the University of Toronto [9]. Additionally, docetaxel is being studied with the CD40 agonist KK2269 in a trial for solid tumors sponsored by Kyowa Kirin [10]. A clinical trial in Shanghai (China) is evaluating efficacy and safety of a four-drug combination including etoposide for small cell lung cancer [11], while another at the Tang-Du Hospital (China) is investigating liposomal irinotecan combined with cisplatin/carboplatin for SCLC patient [12]. Figure 1 shows the chemical structures of four chemotherapeutic drugs broadly utilized in cancer treatment. These agents highlight the different sources of oncological leads, including one isolated directly from nature, one semi-synthetic derivative, and two fully synthetic compounds representing widely used chemical groups (alkylating agents and antimetabolites).

While modern drug discoveries (including molecular docking, structural modeling, and virtual screening) have revolutionized the field, they cannot substitute for the unparalleled chemical diversity provided by the natural world. Nature provides a level of structural complexity that remains indispensable for the development of new anticancer candidates. In fact, it is estimated that over 80% of plant species have yet to be investigated for their pharmacological properties, suggesting that nature still holds untapped potential for the identification of novel anticancer compounds [13,14]. Inspired by the successful historical large-scale screening programs that led to the discovery of landmark drugs such as paclitaxel [15], and given the rich biodiversity of Andalusia (southern part of the Iberian Peninsula) [16], we initiated a research project several years ago to screen the region’s flora for plant species with selective anticancer potential. This process presents significant logistical challenges, including coordinating sample collection with plant life cycles, preparing extracts rapidly to prevent the degradation of active compounds, and executing the subsequent in vitro assays. Consequently, the screening has been performed in stages; to date, we have evaluated and reported on more than 160 species [17,18,19], focusing primarily on Western Andalusia. The strategic objective of this stage is to provide a comprehensive database of bioactivity, including both active and inactive species, to prioritize candidates for future in-depth bio-guided fractionation and chemical characterization. By sharing these results openly, we aim to facilitate the efficient use of resources in natural product research, identifying which species truly justify the high costs of molecular isolation.

In the present work, we continue this effort by evaluating 67 extracts from 59 plant species collected across several regions of Andalusia. The selective anticancer potential of these plant extracts was assessed using human lung cancer cell line A549. The selection of lung cancer as the primary target for this screening is justified by its global impact, being the second most prevalent cancer and leading cause of oncological mortality worldwide. Data from GLOBOCAN 2022 underscores this urgency, reporting approximately 2.5 million new diagnoses and 1.8 million deaths attributed to this disease [1]. Consequently, the A549 cell line was utilized in the present work to ensure methodological continuity with the large-scale screening of the Andalusian flora initiated by our research group [17,18,19].

## 2. Results and Discussion

In the present study, we explored the selective cytotoxic potential of 67 extracts derived from 59 plant species collected in Andalusia (Southern Spain). The plant material evaluated in this study was collected from diverse locations across Andalusia (Tables S1 and S2). This geographic region is recognized as a Mediterranean biodiversity hotspot, characterized by a high rate of endemism and specific climatic conditions [16]. These environmental factors often act as drivers for the biosynthesis of unique secondary metabolites, making the Andalusian flora an exceptional, yet underexplored, reservoir for novel bioactive molecules.

First, the collected plant materials (comprising leaves, flowers, fruits, aerial parts or whole plant depending on the species) were processed and subjected to solvent extraction. Following the extraction process, the extraction yields for all the evaluated samples were calculated and are detailed in Table S2. The yields varied considerably, ranging from 0.3% to 16.6%. This broad range of extraction efficiency is expected and can be attributed to the diverse botanical origins of the samples, the specific plant parts utilized, and their inherent differences in structural matrix and phytochemical density. With this library of 60 extracts established, in vitro selective anticancer activity was evaluated using a dual-cell model: the A549 cell line, representing lung adenocarcinoma, and non-malignant HaCaT keratinocytes as a control for healthy tissue. The resazurin assay was employed to assess cell viability. The A549 cell line was selected to this in vitro screening due to the dominance of NSCLC, which constitutes nearly 85% of all lung cancers, and specifically the adenocarcinoma subtype, which accounts for four out of ten lung cancer cases globally [20]. To determine the selectivity of extracts, the HaCaT human keratinocyte line were used as healthy control [21]. While HaCaT cells originate from epidermal tissue and A549 cells from lung epithelium, both retain epithelial features, making them a suitable comparative model for evaluating selectivity. Furthermore, unlike other normal cells, which are often difficult to maintain due to their requirement for specialized culture media enriched with hormones and growth factors, HaCaT cells can be cultured under standard conditions like those used for cancer cell lines. This consistency minimizes confounding variables in comparative experiments. Normal cells typically undergo a limited number of divisions before senescence, making it challenging to obtain enough for high-throughput screening. Their slow proliferation rate also limits experimental scalability. In contrast, HaCaT cells are spontaneously immortalized, without the need for viral oncogenes such as SV40, which can integrate randomly into the genome and potentially introduce unpredictable phenotypic effects. HaCaT cells maintain stable growth characteristics. Importantly, HaCaT cells, despite being non-malignant, exhibit a high proliferation rate comparable to that of many cancer cell lines. This feature is particularly relevant in the context of cancer treatment, as many cytotoxic drugs preferentially target rapidly dividing cells, both cancer cells and healthy cells. The use of HaCaT cells thus provides a suitable model to evaluate the potential effects of anticancer agents on proliferative non-tumor cells, allowing for a more accurate assessment of therapeutic selectivity and safety [22,23].

**Figure 2:**
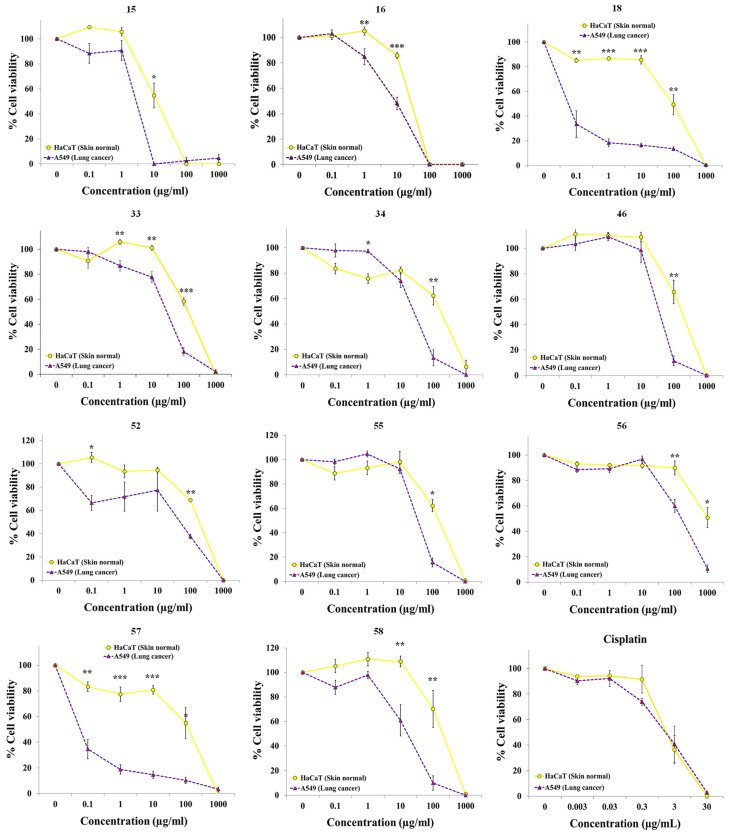
Dose–response curves of selective anticancer extracts (**15**, **16**, **18**, **33**, **34**, **46**, **52**, **55**–**58**) and the anticancer drug cisplatin on A549 lung cancer cells and HaCaT non-malignant cells. Cell viability was quantified with the resazurin assay following a 72 h incubation period. Results are expressed as the mean ± SEM of three or more independent biological replicates. Statistical significance was assessed using a paired *t*-test (levels of significance: * *p* < 0.05, ** *p* < 0.01, *** *p* < 0.001).

The effects of extracts were evaluated by treating A549 and HaCaT cells with different concentrations of the extracts for three days. This extended incubation period was selected to ensure that both cell lines underwent several rounds of replication in the presence of the extracts, facilitating the evaluation of cumulative toxicity. The surviving cell population was then quantified using the resazurin assay. Cisplatin, an anticancer drug used in clinical, was used as positive control under the same experimental conditions. The scientific names of the plants are displayed in Table 1 in alphabetical order, along with botanical family, part of the plant used to elaborate the extract, voucher number, province of origin of the sample, IC_50_ values for each cell line, and selectivity indices. Figure 2 and Supplementary Figures S1–S5 display the concentration-effect curves obtained for all 60 extracts and cisplatin. A wide range of concentrations was used to represent the graphs and allow a fast and easy understanding of the results.

Several extracts (**17**, **32**, **37**, and **43**) were identified as essentially non-toxic under our experimental conditions (Figures S2–S4). These extracts did not reach an IC_50_ threshold in any of the evaluated cell lines, maintaining high cell viability even at the maximum concentration tested (1000 µg/mL).

Other extracts showed cytotoxicity but not selectivity against lung cancer cells (for example, **2**, **4**, **5**, **6**, **9**, **12**, **28**, **31**, **47**, **48** and **53**) and even some others (**1**, **13**, **26**, **41** and **44**) exhibited greater activity against non-malignant cells than against cancer cells (Figures S1–S4). The extract from the aerial part with flowers of *Frankenia laevis* L. (Frankeniaceae, **26**), the extract from the aerial part with flowers of *Helianthemum angustatum* Pomel (Cistaceae, **30**) and the extract from the whole plant of *Scilla peruviana* L. (Hyacinthaceae, **53**) showed a remarkable cytotoxicity against the non-malignant HaCaT cells with IC_50_ values close to 20 µg/mL. The extract from the aerial part with flowers of *Anemone palmata* L. (Ranunculaceae, **3**) and the extract from the aerial part with flowers of *Rumex bucephalophorus* L. (Polygonaceae, **47**) were the most toxic extracts against HaCaT cells, with IC_50_ values 1.3 and 2.1 µg/mL, respectively. To the best of our knowledge, the toxicity of these specific species on human non-malignant cells has not been previously reported, although species within the same genera are known to exert cytotoxicity against various cancer cell lines. A previous study on extracts from *Frankenia laevis* L. reported selective cytotoxicity against human hepatocarcinoma HepG2 cells [24]; however, this selectivity profile was determined by comparing human cancer cells with non-malignant murine cells, rather than a human-derived healthy control. The presence of highly cytotoxic extracts within our local flora underscores the absolute necessity of conducting rigorous safety screenings using appropriate models.

Our results show that some extracts exhibited selective cytotoxicity against the lung cancer cell line A549 (Figure 2, Figures S2 and S4). The extract from the flowers of *Delphinium pentagynum* Lam. (Ranunculaceae, **20**), the extracts from flowers and from leaves of *Dolichandra unguis-cati* (L.) L.G.Lohmann (Bignoniaceae, **23** and **24**), and the extract from flowers of *Clematis flammula* L. (Ranunculaceae, **16**) exhibited modest selectivity, being approximately 2–4-fold more cytotoxic against lung cancer cells (Figure 2 and Figure S2). Ranunculaceae family is a well distributed family of plants worldwide, with around 50 genera, some of them widely distributed as *Anemone*, *Delphinium*, *Clematis* and *Ranunculus* [25]. *Clematis* genus comprises around 295 species [26] and its biological activity might be attributed to the presence of triterpenoids [27,28,29,30]. Some of these triterpenoids, as α-Hederin and D Rhamnose β-Hederin, have shown cytotoxic effects against breast cancer cell lines [31,32]. An ethanol extract from *Clematis cirrhosa* L. showed cytotoxicity against HT-29 colon cancer cell line, inducing cell cycle arrest and apoptosis [33]. Manghaslin and salvadoraside were the main bioactive phenolics identified as contributors to the observed activities. Boehmenan, a lignan extracted from *Clematis armandi* Franch, and different flavonoids from *Clematis aethusifolia* Turcz. were cytotoxic against A549 cells [34,35]. Some of these compounds or different combinations of them could be responsible for the effect observed with the extract from *Clematis flammula* L. in our experiments.

The extract from aerial parts with flowers of *Iberodes linifolia* (L.) M. Serrano, R. Carbajal & S. Ortiz (Boraginaceae, **33**), the extract from the whole plant *Reseda media* Lag. (Resedaceae, **46**), the extract from the whole plant *Saxifraga hirsuta* L. (Saxifragaceae, **52**), the extract from fruits of *Smilax aspera* L. (Smilacaceae, **56**) and the extract from the aerial parts of the endemic *Seseli montanum* subsp. *granatense* (Willk.) C. Pardo (Apiaceae, **55**) were 5–7 times more active against A549 lung cancer cells than against HaCaT non-cancerous cells (Figure 2 and Table 1). To our knowledge, this is the first study to report cytotoxic activity within the genus *Iberodes* (**33**). The extract from *Reseda media* Lag. (**46**) was 5.5-fold times more selective to A549. Another plant from genus *Reseda*, *R. lutea* L., a very well-known mediterranean plant, was reported to have high cytotoxic effect on A549 (lung cancer) and A375 (melanoma) [36,37], an activity that has been attributed to the presence of isothiocyanates [36]. The observed activity of the extract from *Saxifraga hirsuta* (**52**) may be attributed to the presence of phenolic compounds, terpenes, or alkaloids. Several *Saxifraga* species are known to contain alkaloids and triterpenoids that have shown cytotoxic activity against five gastrointestinal cancer cell lines (BGC-823, GBC-SD, CCC-9810, HT-29, and HepG2) [38]. For instance, Liu et al. reported that a water-soluble extract from *Saxifraga stolonifera* inhibited the tumor growth on Lewis lung carcinoma-bearing mice [39]. The authors identified gallic acid, norbergenin, protocatechuic acid, and bergenin as four bioactive components within that extract. Different compounds described in literature or combination of them may participate in the activity found in *Smilax aspera* L. (**56**) fruits extract. For example, other species from this genus were reported to have cytotoxic phenylpropanoid glycosides, flavonoids, tannins, and saponins [40,41,42]. A549 lung cancer cells were 7 times more sensitive to the extract **55** (*Seseli montanum* subsp. *granatense*) than nonmalignant keratinocytes HaCaT. Lignans and a polyacetylenic fatty alcohol have previously been identified as components responsible for the cytotoxic activity in extracts from several *Seseli* species [43,44,45,46].

**Figure 3:**
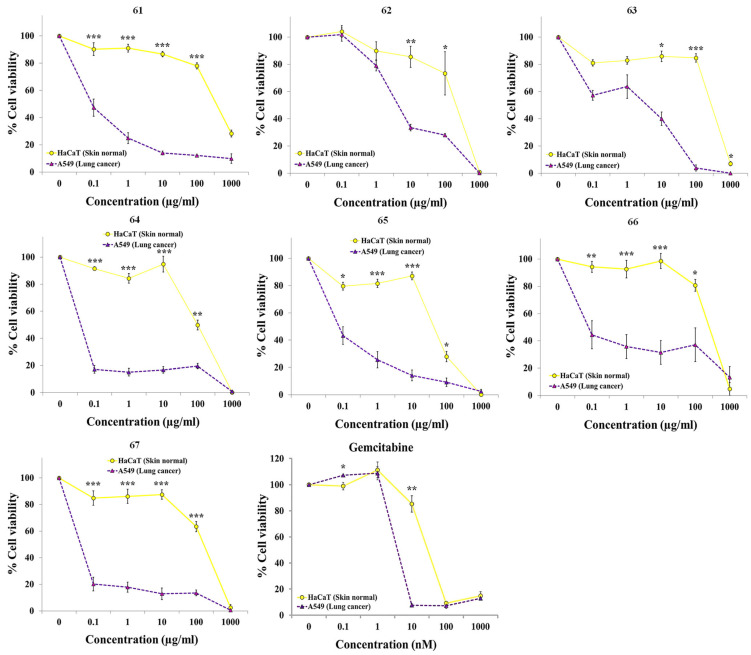
Dose–response curves of plant extracts **61**–**67** and the anticancer drug gemcitabine on A549 lung cancer cells and HaCaT non-malignant cells. Cell viability was quantified with the resazurin assay following a 72 h incubation period. Results are expressed as the mean ± SEM of three or more independent biological replicates. Statistical significance was assessed using a paired *t*-test (levels of significance: * *p* < 0.05, ** *p* < 0.01, *** *p* < 0.001).

The extracts from aerial parts with fruits of *Chamaeiris foetidissima* (L.) Medik. (=*Iris foetidissima* L.) (Iridaceae, **34**), the extract from aerial parts of *Clematis cirrhosa* L. (Ranunculaceae, **15**) and the extract from aerial parts with flowers of *Tordylium officinale* L. (Apiaceae, **58**) displayed very high selective activity against the cancer cells, showing greater selectivity than cisplatin and similar to that of gemcitabine, a drug also used in clinical practice (Figure 2 and Figure 3). We and other authors have reported that Iridaceae family, particularly *Iris* species, is a potential source of anticancer compounds [17,47,48]. In this work, our extract from *Chamaeiris foetidissima* (**34**) was almost 9 times more cytotoxic against the cancer cell lines. Phenolic acids, flavones, flavonols and triterpenoids can be involved in this activity [49,50]. As mentioned previously, triterpenoids, lignans and flavonoids may participate in the activity observed with extracts from the genus *Clematis*. The extract from *Tordylium officinale* L. (**58**) was almost 10-fold times more selective to A549 cancer cells. This could be attributed to the presence in this genus of cytotoxic coumarins [51].

A particularly striking finding was that two extracts from the Thymelaeaceae family, **18** from the leaves of *Daphne gnidium* L. and **57** from the aerial parts of *Thymelaea hirsuta* (L.) Endl., displayed very high cytotoxicity against A549 cancer cells. Even at the lowest concentration tested (0.1 µg/mL), the viability of A549 cells was less than 40%, whereas the viability of non-malignant cells remained above 90% at concentrations up to 10 µg/mL (Figure 2). According to these results, the cytotoxic activity of both extracts was remarkably specific, showing over 1000-fold greater potency against the lung cancer cells compared to the non-cancerous cells. Previously, we reported that an extract from *Daphne laureola* L., another species belonging to Thymelaeaceae family, also showed high selectivity for lung cancer cells [19]. Therefore, we decided to further explore other plants from this family through targeted screening.

One species of *Daphne* and five species of *Thymelaea* were collected to continue this study (Tables S1 and S2). These species were selected based on their accessibility for collection in parks and nature reserves. Extracts were prepared according to our established methodology and subsequently assayed for selective anticancer activity using the resazurin assay. Although all tested extracts showed significant selective activity against A549 lung cancer cells (Figure 3 and Table 2), the extracts from *Thymelaea elliptica* (Boiss.) Endl. (**62**), *Thymelaea granatensis* (Pau) Lacaita (**63**) and *Thymelaea lythroides* Barratte & Murb. (**65**) were the least active and selective. Correspondingly, their IC_50_ values against A549 were the highest of the extracts, with values greater than 0.1 µg/mL. In contrast, the extracts from *Thymelaea lanuginosa* (Lam.) Ceballos & C.Vicioso (**64**) and *Thymelaea tartonraira* subsp. *austroiberica* Lambinon (**66** and **67**) were the most potent and selective against A549 cells. Interestingly, all extracts from both genera displayed greater selectivity than gemcitabine, as they remained non-cytotoxic to normal cells across a broader concentration range while retaining activity against cancer cells. Given that these are crude extracts, the observed activity suggests the presence of constituents with significant potency.

**Figure 4:**
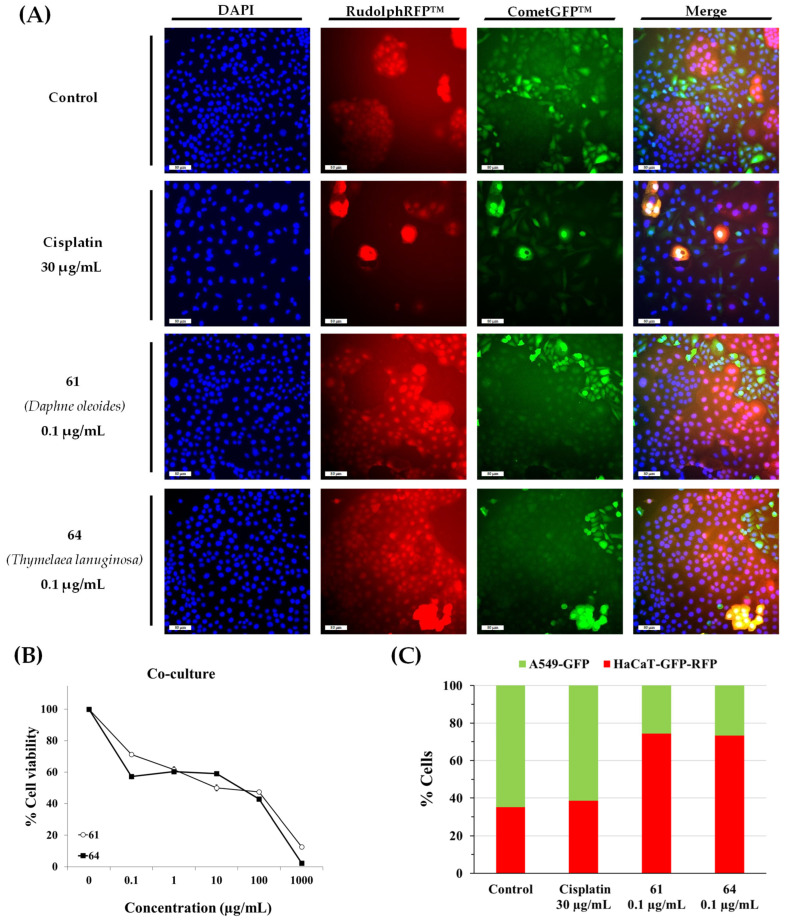
Cytotoxic activity of plant extracts **61**, **64** and cisplatin on HaCaT-GFP-RFP non-malignant cells and A549-GFP lung cancer cells. Following a 72 h treatment of the cocultured populations, cell viability was assessed with the resazurin assay. Subsequently, cell fixation and DAPI-mediated DNA staining were performed. Fluorescence imaging was conducted using a Nikon Eclipse Ti-E microscope at 20× magnification. (**A**) Illustrative photographs. Nuclei were stained with DAPI (blue fluorescence). The A549-GFP cells are identified by green fluorescence (CometGFP expression), while HaCaT-GFP-RFP cells exhibit dual-color labeling (green and red through the co-expression of CometGFP and RudolphRFP). (**B**) Cell Viability and (**C**) differential quantification of each cell type. Data are presented as mean ± SEM from at least two independent biological replicates.

To continue the study, we selected the extracts from *Daphne oleoides* (**61**) and *Thymelaea lanuginosa* (**64**). The extract from *D. oleoides* showed less cytotoxicity against the non-malignant cell line than the extract from *D. gnidium*. The extract from *T. lanuginosa* was one of the most potent and selective and *T. lanuginosa* is the most unknown species. To validate the selective anticancer potential of the selected extracts, a co-culture model was established. Co-cultures were established by seeding A549-GFP and HaCaT-GFP-RFP cells together in the same wells at equal cell numbers (1:1 ratio) in 96-well plates. Following a 72 h exposure to cisplatin or extracts **61** and **64**, viability was quantified with the resazurin assay. Finally, cells were fixed and analyzed using fluorescence microscopy (Figure 4). In untreated control groups, A549-GFP cells exhibited a higher proliferation rate, becoming the dominant population (64.8% vs. 35.2% for HaCaT-GFP-RFP). A comparable distribution was noted in cisplatin-treated samples (61.5% A549-GFP and 38.5% HaCaT-GFP-RFP); although the total cell density was reduced, the drug showed no preference for either cell type, affecting both lines to a similar extent. In contrast, the dominant cell population was reversed in the case of samples treated with both extracts. The cancer cell population decreased by more than half in the samples treated with **61** (25.7% A549-GFP versus 74.3% HaCaT-GFP-RFP). Similar results were observed in the samples treated with **64** (26.6% A549-GFP versus 73.4% HaCaT-GFP-RFP). These results suggest that both extracts have selective anticancer activity, reducing the A549 cancer cell population without significantly harming non-malignant HaCaT cells.

**Figure 5:**
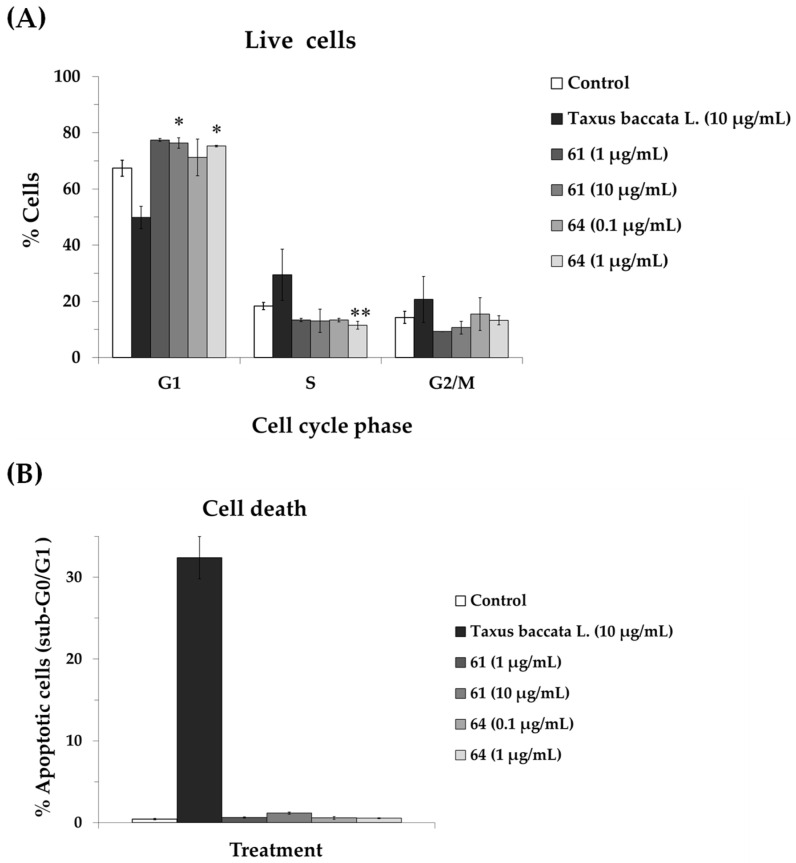
Effects of extract **61** (*Daphne oleoides*), **64** (*Thymelaea lanuginosa*) and *Taxus baccata* on cell cycle distribution in A549 cells following a 72 h exposure. Post-treatment, DNA content was quantified via flow cytometry using propidium iodide staining. (**A**) Analysis of the cell-cycle profile in viable populations. (**B**) Quantification of the lethal fraction (dead cells), defined by a sub-G0/G1 DNA content (<2 N). Results are expressed as mean ± SEM of a minimum of two independent biological replicates. Statistical significance was determined by a paired *t*-test (* *p* < 0.05, ** *p* < 0.01) compared to untreated cells.

To investigate the mechanisms driving the growth-inhibitory effects of extracts **61** and **64**, their impact on the cell cycle distribution of A549 cells was evaluated. An extract from the leaves of *Taxus baccata* L. (Taxaceae), was employed as a positive control due to its characteristic taxane content (mitotic inhibitor) [15,17]. A549 cells were exposed to extracts for 72 h and cell cycle distribution was determined by flow cytometry. As illustrated in Figure 5, *T. baccata* treatment led to a pronounced depletion of the G1 population, coupled with a significant accumulation of cells in the S and G2/M phases, alongside an increase in the sub-G0/G1 (apoptotic) fraction. In contrast, extracts **61** and **64** induced a distinct regulatory pattern, characterized by an expansion of the G1 phase population and a concomitant reduction in the S and G2/M proportions. Neither extract triggered a significant rise in the sub-G0/G1 population, suggesting that their antiproliferative activity is primarily mediated by cell cycle arrest rather than the induction of cytotoxicity. Furthermore, in contrast to the *T. baccata* extract, which exhibited a similar disruption of the cell cycle in both malignant and non-malignant cells, extracts **61** and **64** induced no significant alterations in the cell cycle profiles of HaCaT cells (Figure S6). This clear divergence in the response of healthy cells reinforces the highly selectivity of the studied extracts compared to conventional cytotoxic agents. Similar results have been observed for other species belonging to these genera or for their active constituents [52,53,54,55,56]. Sadeghi et al. [52] reported that the treatment of leukemia cells (K562) for 48 h with an extract of *Daphne mucronata* Royle increased the number of cells in G1 phase. In another study [55], an ethyl acetate extract of *Daphne altaica* Pall. increased the percentage of apoptotic cells and cell cycle arrest in esophageal carcinoma cells (Eca-109). An extract of *Thymelaea hirsuta* L. also induced cell cycle arrest and apoptosis in colorectal cancer cells [57]. Daphnane diterpenoids and flavonoids isolated from several *Daphne* species have been shown to induce cell cycle arrest through several mechanisms [53,54,56,58,59,60] including the regulation of the PI3K/Akt/mTOR signaling pathway, suppression of c-Myc expression, and upregulation of p21.

**Figure 6:**
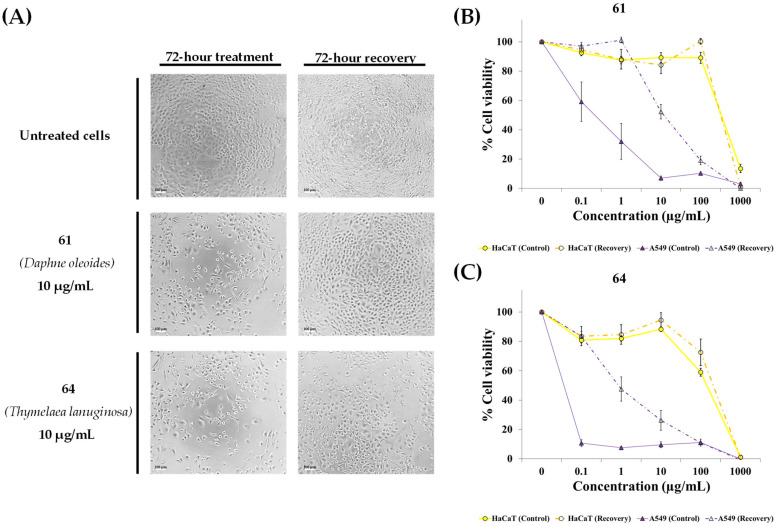
Effect of extracts **61** and **64** on the viability of A549 and HaCaT cells. Cells were treated for 72 h and allowed a 72 h recovery period after treatment removal. Cell viability was quantified with the resazurin assay. (**A**) Representative phase-contrast photographs captured at 10× magnification showing A549 cell density and morphology. (**B**,**C**) Cell viability profiles after 72 h of continuous treatment with extracts (solid lines) compared to a corresponding set of cells allowed to recover in drug-free medium for an additional 72 h (dashed lines). Data represent mean ± SEM from at least three independent biological replicates.

To further investigate the growth inhibition induced by extracts 61 and 64, a recovery assay was performed. A549 and HaCaT cells were treated for 72 h, and their cell viability was measured. Subsequently, the treatment was removed, and the cells were allowed to recover in fresh medium for an additional 72 h. Finally, cell viability was quantified again using resazurin assay. As shown in Figure 6, cells treated with concentrations below 10 µg/mL of extracts showed a significant recovery in metabolic activity and growth after the wash-out period. This evidence, combined with the lack of a sub-G0/G1 population in the flow cytometry data, suggest that these extracts may induce a reversible cell cycle arrest rather than cell death at these concentrations. The comparative analysis of the concentration-response curves reveals that the anticancer selectivity of these extracts is highly dose-dependent. A significant therapeutic window was observed in the ng/mL to low µg/mL range, where the extracts effectively induced a reversible growth arrest (cytostasis) specifically in the malignant A549 cells, without affecting the viability of the non-malignant HaCaT cells. This selectivity, however, disappears at the highest tested concentration (1000 µg/mL), where irreversible cytotoxicity occurs in both cell types. These results suggest that the most active bioactive constituents in these species may act as cytostatic agents. As the dosage increases, less potent secondary metabolites may reach a toxic threshold, leading to the observed lack of recovery at the highest concentrations.

To determinate whether extracts **61** and **64** were also active against other types of cancer, a panel of human cancer cell lines from different tissue origins was evaluated. This panel included bladder cancer (T24), ovarian carcinoma (SK-OV-3), melanoma (MeWo), triple-negative breast cancer (MDA-MB-231) and gastric cancer (KATO-III), assessed alongside the non-malignant HaCaT cells. Following a 72 h exposure to the extracts **61** and **64**, or gemcitabine (positive control), cell viability was quantified with the resazurin assay (Table 3). Both extracts maintained substantial cytotoxicity against the breast and bladder cancer cell lines, albeit requiring higher half-maximal inhibitory concentrations (IC_50_ values ranging from 9.6 to 106.3 µg/mL). Both extracts achieved a selectivity index greater than 4.0 for the T24 and MDA-MB-231 cells, indicating a significant preference for these malignant cells over the non-cancerous HaCaT cells. Gemcitabine failed to exhibit any selectivity against MDA-MB-231 cells and reached a 4-fold selectivity for the T24 cells. However, the targeted selectivity was entirely lost in the MeWo melanoma cells. Furthermore, a divergent susceptibility profile was observed between the gastric and ovarian cancer cells lines: extract **61** exhibited preferential toxicity towards KATO-III over SK-OV-3, whereas extract **64** displayed the inverse trend. Collectively, these findings underscore that the therapeutic window of these extracts is highly dependent on the specific tumor type. The differential activity observed across theses cancer cell lines may be attributed to intrinsic differences in p53 status, redox homeostasis, epithelial–mesenchymal phenotype, and the predominance of distinct survival signaling pathways such as MAPK, PI3K/AKT, and c-MET.

The activity profile of the extracts is consistent with previously reported mechanisms for bioactive compounds from the Thymelaeaceae family. This family is composed of about 50 genera [61]. The main genera in this family are *Daphne* and *Thymelaea*. The genus *Thymelaea* is a monophyletic group considered a sister group to the genus *Daphne* [62]. Worldwide, *Daphne* and *Thymelaea* comprise approximately 92 and 34 accepted species, respectively [63,64]. The Thymelaeceae family is known for its potential cytotoxic activity, and plants within this family have been researched for their biological activities [65,66,67,68,69]. Some plants of both genera have been used as traditional remedies in the ethnopharmacology of China, Mediterranean, and North African countries [66,70]. For example, a decoction of the aerial parts of *Thymelaea lythroides* Barratte & Murb. has been traditionally used for the treatment of uterus cancer in Morocco [71]. Previous phytochemical studies of these genera have shown the presence of acrylates, coumarins, cromenes, cyclohexanones, diterpenenoids, esterols, flavonoids, lignans, phenolic compounds, sesquiterpenes [65,66,70,72,73,74,75]. Daphnane-type diterpenoids are the main biologically active constituents, exhibiting a wide range of pharmacological activities such as anticancer, antiinflammatory, and anti-HIV effects. There are five known groups of daphanane diterpenes: daphnetoxins, 12-hydroxydaphnetoxins, 1-alkydaphnanes, genkwanines, and resiniferonoids [65,72,73]. The most cytotoxic are those with a special orthoester group present in c-9, c-13, and c-14 [65,72,73]. While most daphnane diterpenes have been identified from the genus *Daphne*, the diversity of these compounds in the genus *Thymelaea* remains poorly understood [65,66,73,76]. For example, daphnane diterpenes isolated from the flowers of *Daphne genkwa* Siebold & Zucc. were highly cytotoxic against A549, with low activity against MRC-5 normal lung epithelial cells [77]. Yuanhuacine, one of these compounds, was also studied in H1993 lung carcinoma cells, affecting AMPK/mTORC2 signaling pathway and actin cytoskeleton organization [78]. Other daphnane diterpenes, such as yuanhadine, yuanhualine, yuanhuahine and yuanhuagine altered cell cycle in A549 cells by interfering with signaling pathways (Akt/mTOR, EGFR or Src pathways) [53,79]. Several extracts of different *Daphne* species or their constituents have also shown activity against other types of cancer [68,69,80,81,82,83,84,85,86]. For example, yuanhuatine selectively induces mitochondrial apoptosis in breast cancer cells (MCF-7) [85] and yuanhuacine induces cell cycle arrest in bladder cancer cells (T24T, a metastatic variant of the T24 cell line) by upregulating p21 protein expression levels [86].

Furthermore, it has been widely suggested that the selectivity of many phytochemicals against cancer cells may stem from the elevated basal levels of reactive oxygen species (ROS) characteristic of cancer cells, which renders them more susceptible to pro-oxidant stimuli [87,88,89]. To investigate whether this mechanism was responsible for the activity of extracts **61** and **64**, we performed a rescue experiment using the antioxidants N-acetylcysteine (NAC) and catalase. NAC acts as a direct ROS scavenger and a precursor for glutathione synthesis, thereby mitigating intracellular oxidative stress, while enzyme catalase neutralizes hydrogen peroxide (H_2_O_2_). A549 were exposed to these extracts in the presence or absence of the antioxidants for 72 h and cell viability was quantified by the resazurin assay (Figure S7). While both antioxidants successfully rescued cell viability in the presence of H_2_O_2_ (positive control), they failed to mitigate the effects induced by extracts **61** and **64**. These findings suggest that ROS generation may not play a dominant role in the observed selective activity of these extracts. Instead, they point toward a potentially more specific, ROS-independent anticancer mechanism.

The lack of protective effect of NAC and catalase, together with the cytotoxic activity observed for both extracts across different cancer cell lines, strongly suggests that their mechanism of action is not mediated by oxidative stress. As described above, daphnane-type diterpenoids present in *Daphne* species have been shown to affect multiple signaling pathways involved in cell proliferation [53,78,79,86]. The significant selectivity towards MDA-MB-231 (triple-negative breast cancer) and T24 (bladder cancer) suggests that the bioactive phytocompounds of these extracts may target non-hormonal signalling pathways, such as PI3K/AKT or RAS/MAPK signalling, upon which these aggressive phenotypes heavily rely. Furthermore, the differential sensitivity observed between KATO-III (gastric cancer) and SK-OV-3 (ovarian cancer) cells points to the possibility that the extracts may contain distinct bioactive compounds targeting different molecular pathways, such as c-MET-dependent signalling in gastric cancer cells or PI3K/AKT pathways in ovarian carcinoma. In contrast, the lack of selective activity in MeWo melanoma cells may reflect their intrinsic resistance to multiple cytotoxic mechanisms, including the high expression of ABC efflux transporters. Future phytochemical characterization and mechanistic studies will be essential to identify the precise molecular targets of these plants.

## 3. Materials and Methods

### 3.1. Plant Material

The plant material for this paper was collected by V. Jiménez-González between July 2018 and May 2022 in Cadiz, Córdoba, Granada, Jaén, Huelva, and Sevilla (Andalusia, Spain). Samples of vegetal origin (9–90 g) were collected following the guidelines for individual and environmental protection. We deposited voucher specimens for each plant in the herbarium of the University of Seville located at the Center for Research, Technology and Innovation (CITIUS II Celestino Mutis). Scientific name of species collected, plant part used, and voucher numbers are displayed in Table 1. The coordinates of collection are shown in Supplementary Table S1.

### 3.2. Preparation of the Extracts

Plant extracts were prepared within the first few hours following collection. Extraction was performed using a solvent mixture (100 to 200 mL of the mixture solvents: ethanol/ethyl acetate/water, 1:1:1 *v*/*v*/*v*) in an ultrasound water bath for 1 h at 60 °C. To ensure maximum efficiency, the ultrasound unit was operated at its maximum power output throughout the process.

After the extraction, solid particles, ethanol and ethyl acetate and water were sequentially eliminated by vacuum filtration, rotary evaporation and lyophilization respectively. After 72 h of lyophilization, the dried extracts were obtained. The final percentage yields (%) of the extracts prepared for our screening are summarized in Table S2.

Amber glass bottles were used to store the different extracts and preserved in a dark and cool room in the Department of Pharmacology of University of Seville. The first cell viability assay was performed in the next month after preparation to avoid any degradation of the compounds. A 100 mg/mL stock solution was prepared with DMSO, and the working solutions were prepared in the culture medium. The final DMSO concentration in all experimental groups was maintained below 1% (*v*/*v*), being non-toxic to the cells.

### 3.3. Drugs and Reagents

The following reagents were used: ECOSURF™ EH-9, resazurin, and ribonuclease A (Sigma Aldrich, St. Louis, MO, USA); gemcitabine (Pfizer S.L., Madrid, Spain); cisplatin and DAPI (Thermo Scientific Acros Organics, Waltham, MA, USA); and propidium iodide (Panreac Applichem, Darmstadt, Germany).

### 3.4. Cell Lines

The human cell lines A549 (non-small-cell lung cancer), KATO III (gastric cancer), MeWo (melanoma), SK-OV-3 (ovarian cancer), T24 (bladder cancer), and the non-malignant HaCaT skin cells [21] were all purchased from Cytion (Heidelberg, Germany). The MDA-MB-231 breast cancer line was provided by the American Type Culture Collection (ATCC, Manassas, VA, USA). Additionally, HaCaT-GFP-RFP and A549-GFP cells were utilized as previously detailed [17].

All cultures were maintained in Dulbecco’s Modified Eagle Medium (DMEM) containing L-glutamine, 4.5 g/L D-glucose, 10% fetal bovine serum, and 100 U/mL/100 µg/mL of penicillin/streptomycin. Standard culture conditions (37 °C, humidified atmosphere, 5% CO_2_) were applied. Unless specified, all culture consumables were obtained from Thermo Fisher Scientific or Biowest (Nuaillé, France).

### 3.5. Cell Viability Assay

The cytotoxic effect was evaluated using the resazurin assay. This colorimetric method relies on the metabolic capacity of living cells to convert the non-fluorescent blue dye (resazurin) into the pink, fluorescent product (resorufin) via redox reactions. Since this conversion is directly coupled to cellular metabolic activity, the resulting color intensity serves as a proxy for the number of viable cells. Briefly, cells were seeded at a density of 3000 and 5000 cells/well in 96-well plates. After a 24 h stabilization period, cells were treated for 72 h with varying concentrations of plant extracts or anticancer drugs. Post-treatment, a washing step with phosphate-buffered saline (PBS) was performed, followed by the addition of 150 μL of resazurin solution (20 μg/mL in medium). Following a 4–5 h incubation, the metabolic reduction of resazurin to resorufin was quantified by measuring absorbance at 540 and 620 nm using an iMark microplate reader (Bio-Rad Laboratories Inc., Hercules, CA, USA).

Cell viability was calculated as a percentage relative to untreated cells. Results represent the mean ± standard error of the mean (SEM) from at least three separate experiments. To determine the selectivity of the extracts, the selectivity index (S.I.) was calculated. The S.I. reflects the average of the individual IC_50_ ratios (non-malignant HaCaT/malignant A549) obtained in each independent experiment.

### 3.6. Co-Culture Assay

HaCaT-GFP-RFP and A549-GFP were co-cultured (3000 per well for each cell line) in 96-well plates. After a 24 h stabilization period, cells were treated for 72 h with varying concentrations of plant extracts or anticancer drug. Post-treatment, a washing step with phosphate-buffered saline (PBS) was performed, followed by the fixation with 70% cold ethanol and DNA staining with DAPI. Images were captured using a Nikon Eclipse Ti-E epifluorescence microscope (Nikon Europe B.V, Amstelveen, The Netherlands) at 20× magnification. Quantitative analysis of the co-culture was performed through manual cell counting to overcome the limitations of automated software in segmenting overlapping or clustered cells. For each well, between 150 and 350 cells were analyzed. The total cell population was determined by counting DAPI-stained nuclei. HaCaT-GFP-RFP cells were specifically identified by their red fluorescence (RFP). The A549-GFP subpopulation was subsequently calculated by subtracting the number of RFP-positive cells from the total DAPI-stained count. This manual approach ensured the accurate discrimination of individual cells within dense clusters.

### 3.7. Cell Cycle

For cell cycle analysis, cells were plated in 6-well plates at a density of 200,000 cells/well and incubated for 24 h to ensure attachment. Following medium renewal, treatments with plant extracts were applied for a 72 h period. Both the supernatant and trypsin-detached cells were subsequently harvested, centrifuged (300× *g*, 4 min, 4 °C), and rinsed twice using ice-cold PBS. Cell fixation was achieved by incubating the pellets in 70% ethanol at 4 °C for one hour. After an additional PBS wash, samples were treated with a staining cocktail (30 µg/mL propidium iodide, 0.1% Tween 20, 10 µg/mL ribonuclease A and 0.1% ECOSURF™ EH-9) for 60 min at 4 °C. DNA content and cell cycle distribution were then analyzed using a Beckman Coulter CYTOMICS FC500 flow cytometer (High Wycombe, UK).

### 3.8. Statistical Analysis

Statistical evaluations were performed using Microsoft Excel 365 (Microsoft Corp., Redmond, WA, USA). Data were analyzed using a paired, two-tailed Student’s *t*-test to determine differences between experimental groups. Statistical significance was defined by a *p*-value threshold of 0.05; results exceeding this value (*p* ≥ 0.05) were considered non-significant and are not marked with symbols. Significant differences are indicated in the figures as follows: * (*p*-value < 0.05), ** (*p* ≤ 0.01), and *** (*p* ≤ 0.001).

## 4. Conclusions

This study expanded a screening project aimed at identifying plant species from Andalusia with potential selective anticancer activity. Herein, we evaluated the selective anticancer activity of 59 plant species. Extracts from *Chamaeiris foetidissima* (L.) Medik. (Iridaceae), *Clematis cirrhosa* L. (Ranunculaceae), *Daphne gnidium* L. (Thymelaeaceae), *Iberodes linifolia* (L.) M.Serrano, R. Carbajal & S. Ortiz (Boraginaceae), *Reseda media* Lag. (Resedaceae), *Saxifraga hirsuta* L. (Saxifragaceae), *Seseli montanum* subsp. *granatense* (Willk.) C. Pardo (Apiaceae), *Smilax aspera* L. (Smilacaceae), *Thymelaea hirsuta* (L.) Endl. (Thymelaeaceae), and *Tordylium officinale* L. (Apiaceae) demonstrated significant selective cytotoxicity against A549 lung adenocarcinoma cells. The Thymelaeaceae family, particularly the genera *Thymelaea* and *Daphne*, emerged as the most promising candidates for further development. The extracts from *D. gnidium*, *D. oleoides*, *T. hirsuta*, *T. lanuginosa* and *T. tartonraira* were over 1000 times more active against A549 lung cancer cells than against HaCaT non-malignant cells.

A more in-depth study was carried out on the selective anticancer activity of the extracts from *D. oleoides* (the least cytotoxic against non-malignant cells) and *T. lanuginosa* (the most potent and selective against lung cancer cells). Our results in a co-culture model with A549-GFP and HaCaT-GFP-RFP cells confirm that the extracts from *D. oleoides* and *T. lanuginosa* possess a high degree of selectivity, effectively sparing non-malignant cells while targeting cancer cells. At low concentrations, the antiproliferative effect of these extracts may be mediated by a reversible arrest in the G1 phase of the cell cycle. Their antiproliferative effects are not mediated by the induction of acute oxidative stress, as confirmed by the lack of rescue effect with antioxidants (NAC and catalase).

In addition, the observed activity was not limited to lung cancer cells. The extracts showed activity in several cancer cell lines beyond lung cancer, though this activity was not always associated with selective cytotoxicity. They also exhibited selective cytotoxic effects against triple-negative breast (MDA-MB-231) and bladder (T24) cancer cells, but not to melanoma (MeWo) cells.

Despite these encouraging results, this study represents an initial screening approach aimed at identifying biologically active extracts rather than providing a comprehensive mechanistic characterization. A key limitation is the lack of phytochemical characterization, highlighting the need for bioassay-guided fractionation to isolate and identify the compounds responsible for the observed effects. Additionally, future research should expand the panel of both cancerous and non-malignant cell lines to allow a more accurate assessment of selectivity and therapeutic window. Finally, further molecular studies are required to elucidate the underlying signalling pathways, together with validation in in vivo tumor models to confirm the therapeutic potential of these plant species.

## Figures and Tables

**Table 1:** Pharmacological and ethnobotanical profile of the 60 studied extracts, including species, family, plant part used, voucher numbers, collection sites, IC_50_ values and Selectivity Index (S.I.).

Extract	Species	Family	Plant Part Used	Voucher Number (SEV)	Collection Site	IC_50_ (Mean ± SEM, µg/mL)		S.I. (Mean ± SEM)
						A549 (Cancer)	HaCaT (Non-Malignant)	
**1**	*Acanthus mollis* L.	Acanthaceae	Aerial part with flowers	289720	Cordoba	>1000	311.3 ± 81.0	0.4 ± 0.2
**2**	*Acanthus mollis* L.	Acanthaceae	Fruits	289720	Cordoba	335.7 ± 33.3	275.5 ± 22.2	0.9 ± 0.1
**3**	*Anemone palmata* L.	Ranunculaceae	Aerial part with flowers	290026	Huelva	0.8 ± 0.3	1.3 ± 0.6	1.5 ± 0.7
**4**	*Anogramma leptophylla* (L.) Link	Hemionitidaceae	Aerial part	289717	Cordoba	501.4 ± 172.2	303.9 ± 23.2	0.7 ± 0.3
**5**	*Antirrhinum cirrhigerum* (Welw. ex Ficalho) Rothm.	Veronicaceae	Aerial plant with flowers	289239	Sevilla	640.7 ± 140.2	528.9 ± 92.5	0.8 ± 0.2
**6**	*Asphodelus fistulosus* L.	Asphodelaceae	Aerial parts with flowers	288083	Sevilla	272.4 ± 23.5	405.9 ± 23.1	1.0 ± 0.4
**7**	*Asphodelus ramosus* L.	Asphodelaceae	Leaves	290005	Sevilla	201.8 ± 7.9	263.1 ± 12.3	1.3 ± 0.1
**8**	*Asphodelus ramosus* L.	Asphodelaceae	Flowers	290005	Sevilla	>1000	>1000	N.D.
**9**	*Ballota hirsuta* Benth.	Lamiaceae	Aerial part with flowers	289718	Cordoba	369.0 ± 49.0	336.8 ± 9.7	1.0 ± 0.1
**10**	*Bryonia cretica* subsp. *dioica* (Jacq.) Tutin	Cucurbitaceae	Aerial part	289999	Sevilla	>1000	>1000	N.D.
**11**	*Cardamine hirsuta* L.	Brassicaceae	Whole plant	289998	Sevilla	225.7 ± 17.5	260.0 ± 37.4	1.2 ± 0.2
**12**	*Carpobrotus edulis* (L.) N.E.Br.	Aizoaceae	Flowers	290024	Huelva	76.1 ± 19.4	44.1 ± 7.1	0.7 ± 0.2
**13**	*Carpobrotus edulis* (L.) N.E.Br.	Aizoaceae	Leaves	290024	Huelva	226.3 ± 30.0	104.0 ± 32.9	0.4 ± 0.1
**14**	*Cerinthe major* L.	Boraginaceae	Aerial parts with flowers	288089	Sevilla	170.8 ± 48.2	167.6 ± 52.4	1.5 ± 0.8
**15**	*Clematis cirrhosa* L.	Ranunculaceae	Aerial part	290018	Cádiz	2.1 ± 0.7	13.3 ± 3.6	9.1 ± 7.3
**16**	*Clematis flammula* L.	Ranunculaceae	Flowers	289722	Cordoba	7.4 ± 2.1	28.1 ± 1.0	4.3 ± 0.7
**17**	*Crambre filiformis* Jacq.	Brassicaceae	Aerial part with flowers	289716	Cordoba	>1000	>1000	N.D.
**18**	*Daphne gnidium* L.	Thymelaeaceae	Leaves	288067	Sevilla	<0.1	106.7 ± 32.4	>1000
**19**	*Delphinium pentagynum* Lam.	Ranunculaceae	Aerial part with flowers	289721	Cordoba	489.4 ± 76.0	525.3 ± 40.3	1.2 ± 0.2
**20**	*Delphinium pentagynum* Lam.	Ranunculaceae	Flowers	289721	Cordoba	30.8 ± 4.8	211.3 ± 29.8	3.7 ± 1.6
**21**	*Dioscorea communis* (L.) Caddick & Wilkin	Dioscoreaceae	Aerial part	290002	Sevilla	273.9 ± 55.3	321.7 ± 29.5	1.4 ± 0.4
**22**	*Dipcadi serotinum* (L.) Medik.	Hyacinthaceae	Whole plant	289805	Huelva	347.9 ± 34.9	261.4 ± 31.2	0.8 ± 0.1
**23**	*Dolichandra unguis-cati* (L.) L.G.Lohmann *	Bignoniaceae	Flowers	290029	Sevilla	246.3 ± 38.2	542.2 ± 23.6	1.9 ± 0.8
**24**	*Dolichandra unguis-cati* (L.) L.G.Lohmann *	Bignoniaceae	Leaves	290029	Sevilla	86.7 ± 23.6	249.8 ± 10.2	2.6 ± 1.1
**25**	*Foeniculum vulgare* Mill.	Apiaceae	Aerial part	289989	Sevilla	309.1 ± 32.1	457.7 ± 112.9	1.4 ± 0.2
**26**	*Frankenia laevis* L.	Frankeniaceae	Aerial part with flowers	290022	Cádiz	92.9 ± 57.1	20.9 ± 3.4	0.5 ± 0.2
**27**	*Geranium purpureum* Vill.	Geraniaceae	Aerial part	289715	Cordoba	36.9 ± 4.1	61.7 ± 4.1	1.8 ± 0.2
**28**	*Gladiolus × byzantinus* Mill.	Iridaceae	Aerial parts with flowers	289236	Sevilla	219.2 ± 44.7	188.5 ± 15.2	1.0 ± 0.2
**29**	*Globularia spinosa* L.	Globulariaceae	Aerial part	289794	Jaén	55.1 ± 25.9	58.7 ± 15.9	0.7 ± 0.4
**30**	*Helianthemum angustatum* Pomel	Cistaceae	Aerial part with flowers	289991	Sevilla	64.1 ± 25.0	24.8 ± 2.6	0.8 ± 0.3
**31**	*Helianthemum hirtum* (L.) Mill.	Cistaceae	Aerial part with flowers	289992	Sevilla	88.5 ± 31.6	77.7 ± 15.1	0.9 ± 0.1
**32**	*Himantoglossum robertianum* (Loisel.) P.Delforge	Orchidaceae	Aerial part with flowers	290001	Sevilla	>1000	>1000	N.D.
**33**	*Iberodes linifolia* (L.) M.Serrano. R.Carbajal & S.Ortiz	Boraginaceae	Aerial part with flowers	289724	Cordoba	30.6 ± 3.5	139.5 ± 14.4	5.0 ± 0.6
**34**	*Chamaeiris foetidissima* (L.) Medik. (=*Iris foetidissima* L.)	Iridaceae	Aerial part with fruits	290023	Cádiz	25.2 ± 5.0	188.2 ± 59.7	8.9 ± 4.3
**35**	*Jasione montana* L.	Campanulaceae	Aerial part with flowers	289719	Cordoba	491.3 ± 95.3	462.6 ± 57.7	0.8 ± 0.3
**36**	*Lamarckia aurea* (L.) Moench	Poaceae	Whole plant	289243	Sevilla	100.5 ± 16.0	118.4 ± 18.1	1.2 ± 0.1
**37**	*Lepidium didymum* (L.) Sm.	Brassicaceae	Whole plant	289733	Sevilla	>1000	>1000	N.D.
**38**	*Linum appressum* Caball.	Linaceae	Aerial part	289795	Jaén	423.4 ± 53.3	483.6 ± 74.0	1.3 ± 0.3
**39**	*Misopates calycinum* (Lange) Rothm.	Veronicaceae	Whole plant	289238	Sevilla	>1000	855.2 ± 179.0	N.D.
**40**	*Nepeta tuberosa* L.	Lamiaceae	Aerial part with flowers	289726	Cordoba	528.9 ± 114.7	355.1 ± 15.0	0.8 ± 0.2
**41**	*Nepeta tuberosa* L.	Lamiaceae	Roots	289726	Cordoba	>1000	415.0 ± 57.0	N.D.
**42**	*Osyris alba* L.	Santalaceae	Aerial part with flowers	289713	Cordoba	246.6 ± 47.7	273.6 ± 17.1	1.3 ± 0.3
**43**	*Parietaria hirsuta* L.	Urticaceae	Whole plant	288079	Sevilla	>1000	>1000	N.D.
**44**	*Petrosedum forsterianum* (Sm.) Grulich	Crassulaceae	Whole plant	290019	Cádiz	324.8 ± 9.7	130.3 ± 43.7	0.5 ± 0.1
**45**	*Pteridium aquilinum* (L.) Kuhn	Hypolepidaceae	Aerial part	290007	Huelva	319.7 ± 14.9	313.5 ± 22.0	0.7 ± 0.3
**46**	*Reseda media* Lag.	Resedaceae	Whole plant	290014	Huelva	35.2 ± 7.0	140.0 ± 55.7	5.5 ± 2.6
**47**	*Rumex bucephalophorus* L.	Polygonaceae	Aerial part with flowers	290025	Huelva	1.8 ± 0.6	2.1 ± 1.1	0.9 ± 0.3
**48**	*Rumex spinosus* L.	Polygonaceae	Whole plant	288080	Sevilla	363.9 ± 27.1	312.9 ± 4.1	0.9 ± 0.1
**49**	*Ruscus aculeatus* L.	Ruscaceae	Leaves	290009	Huelva	>1000	>1000	N.D.
**50**	*Ruscus aculeatus* L.	Ruscaceae	Fruits	290009	Huelva	208.3 ± 26.4	294.9 ± 23.9	1.5 ± 0.3
**51**	*Ruta montana* (L.) L.	Rutaceae	Aerial part with flowers	290000	Sevilla	143.3 ± 39.0	189.9 ± 22.2	2.2 ± 1.2
**52**	*Saxifraga hirsuta* L.	Saxifragaceae	Whole plant	290003	Sevilla	37.9 ± 11.3	187.8 ± 2.5	6.0 ± 1.4
**53**	*Scilla peruviana* L.	Hyacinthaceae	Whole plant	290021	Cádiz	24.5 ± 4.2	25.2 ± 2.0	1.2 ± 0.4
**54**	*Selaginella denticulata* (L.) Spring	Selaginellaceae	Whole plant	290004	Sevilla	110.3 ± 36.1	150.5 ± 29.2	1.7 ± 0.7
**55**	*Seseli montanum* subsp. *granatense* (Willk.) C. Pardo	Apiaceae	Aerial part	289796	Jaén	36.3 ± 3.9	279.1 ± 46.9	7.5 ± 0.5
**56**	*Smilax aspera* L.	Smilacaceae	Fruits	290010	Huelva	163.9 ± 30.8	>1000	> 5.2
**57**	*Thymelaea hirsuta* (L.) Endl.	Thymelaeaceae	Aerial parts	288069	Cádiz	<0.1	134.7 ± 39.3	>1000
**58**	*Tordylium officinale* L.	Apiaceae	Aerial part with flowers	289723	Cordoba	21.8 ± 8.9	169.0 ± 62.9	9.9 ± 2.4
**59**	*Trachelium caeruleum* L.	Campanulaceae	Leaves	289725	Cordoba	172.6 ± 50.8	261.4 ± 39.4	1.8 ± 0.8
**60**	*Tradescantia pallida* (Rose) D.R.Hunt *	Commelinaceae	Aerial parts	288082	Sevilla	487.1 ± 113.4	>1000	N.D.
	Cisplatin					2.8 ± 1.6	2.3 ± 1.1	0.9 ± 0.1

(*) Non-native species of ornamental origin currently found in wild habitats. Bold numbers represent the specific extract identifier. A Voucher specimen was deposited in the Herbarium of the University of Seville (SEV) for each plant. The selectivity index reflects the average of the individual IC_50_ ratios (non-malignant HaCaT/malignant A549) obtained in each independent experiment; N.D.: not determined.

**Table 2:** Pharmacological and ethnobotanical profile of the studied Thymelaeaceae family, including species, plant part used, voucher numbers, collection sites, IC_50_ values and Selectivity Index (S.I).

Extract	Species	Family	Plant Part Used	Voucher Number (SEV)	Collection Site	IC_50_ (Mean ± SEM, µg/mL)		S.I. (Mean ± SEM)
						A549 (Cancer)	HaCaT (Non-Malignant)	
**61**	*Daphne oleoides* Schreb.	*Thymelaeaceae*	Aerial part with flowers	289192	Cádiz	<0.1	371.2 ± 21.8	>1000
**62**	*Thymelaea elliptica* (Boiss.) Endl.	*Thymelaeaceae*	Aerial part with flowers	289792	Granada	4.4 ± 0.4	198.9 ± 73.1	44.3 ± 14.2
**63**	*Thymelaea granatensis* (Pau) Lacaita	*Thymelaeaceae*	Aerial part	289797	Jaén	5.1 ± 2.4	279.9 ± 15.7	97.5 ± 30.7
**64**	*Thymelaea lanuginosa* (Lam.) Ceballos & C.Vicioso	*Thymelaeaceae*	Aerial part	289222	Sevilla	<0.1	142.2 ± 56.3	>1000
**65**	*Thymelaea lythroides* Barratte & Murb.	*Thymelaeaceae*	Aerial part	289798	Sevilla	0.5 ± 0.3	40.9 ± 4.3	228.9 ± 108.8
**66**	*Thymelaea tartonraira* subsp. *austroiberica* Lambinon	*Thymelaeaceae*	Aerial part	289226	Granada	<0.1	135.0 ± 54.7	>1000
**67**	*Thymelaea tartonraira* subsp. *austroiberica* Lambinon	*Thymelaeaceae*	Bark	289226	Granada	<0.1	163.9 ± 17.0	>1000
	Gemcitabine (nM)					3.8 ± 0.2	28.8 ± 2.9	7.6 ± 0.9

Bold numbers represent the specific extract identifier. A Voucher specimen was deposited in the Herbarium of the University of Seville (SEV) for each plant. The selectivity index reflects the average of the individual IC_50_ ratios (non-malignant HaCaT/malignant A549) obtained in each independent experiment.

**Table 3:** IC_50_ values and selectivity indices (S.I.) of extract **61**, **64** and gemcitabine on a panel of human cell lines.

	Extract 61 (Mean ± SEM)		Extract 64 (Mean ± SEM)		Gemcitabine (Mean ± SEM)	
Cell Line	IC_50_ (µg/mL)	S.I.	IC_50_ (µg/mL)	S.I.	IC_50_ (ng/mL)	S.I.
HaCaT (Non-malignant keratinocytes)	404.2 ± 82.0	-	179.1 ± 28.5	-	8.0 ± 0.1	-
KATO-III (Gastric cancer)	94.1 ± 5.7	3.6 ± 0.8	406.8 ± 23.3	0.5 ± 0.1	6.0 ± 0.3	1.4 ± 0.1
MDA-MB-231 (Breast cancer)	51.0 ± 33.3	12.0 ± 6.3	9.6 ± 2.2	14.3 ± 2.8	338.6 ± 27.0	0.0 ± 0.0
MeWo (Melanoma)	235.3 ± 22.4	1.4 ± 0.4	143.4 ± 25.5	1.3 ± 0.2	5.2 ± 0.3	1.6 ± 0.1
SK-OV-3 (Ovarian cancer)	>1000	0.2 ± 0.1	41.4 ± 3.1	2.6 ± 0.3	387.0 ± 154.9	0.04 ± 0.0
T24 (Bladder cancer)	106.3 ± 16.4	4.4 ± 1.1	53.6 ± 21.4	5.0 ± 1.7	2.0 ± 0.1	4.0 ± 0.2

## Data Availability

The original contributions presented in this study are included in the article/Supplementary Materials. Further inquiries can be directed to the corresponding authors.

## Supplementary Materials

The following supporting information can be downloaded at: https://www.mdpi.com/article/10.3390/ph19040616/s1, Table S1. Geographical coordinates of the collection sites for the botanical species evaluated in this study. Table S2. The extraction yields (%) obtained for each of the botanical extracts evaluated in the present study. Figure S1. Assessment of the differential cytotoxic effects of extracts **1**–**12** on human lung adenocarcinoma (A549) and non-tumorigenic cells (HaCaT). Figure S2. Assessment of the differential cytotoxic effects of extracts **13**–**14**, **17**, **19**–**27** on human lung adenocarcinoma (A549) and non-tumorigenic cells (HaCaT). Figure S3. Assessment of the differential cytotoxic effects of extracts **28**–**32, 35**–**41** on human lung adenocarcinoma (A549) and non-tumorigenic cells (HaCaT). Figure S4. Assessment of the differential cytotoxic effects of extracts **42**–**45, 47**–**51, 53**–**54** and **59** on human lung adenocarcinoma (A549) and non-tumorigenic cells (HaCaT). Figure S5. Assessment of the differential cytotoxic effects of extract **60** on human lung adenocarcinoma (A549) and non-tumorigenic cells (HaCaT). Figure S6. Effects of extract **61** (*Daphne oleoides*), **64** (*Thymelaea lanuginosa*) and *Taxus baccata* on cell cycle distribution in HaCaT cells following a 72 h exposure. Figure S7. Assessment of the contribution of ROS generation to the cytotoxicity of the extract **61** (*Daphne oleoides*), **64** (*Thymelaea lanuginosa*) in A549 cells.
