# Supplementary material for: Screening for Selective Anticancer Activity of Extracts from 59 Plant Species Collected in Southern Spain (Andalusia)
Source: Pharmaceuticals (Basel). 2026 Apr 14;19(4):616. doi: 10.3390/ph19040616 (PMC13118989; doi:10.3390/ph19040616)
Supplement: Supplementary file 1 [file pharmaceuticals-19-00616-s001.zip › pharmaceuticals-4205523-supplementary.pdf]

Article

Screening for Selective Anticancer Activity of Extracts from 59 Plant Species Collected in Southern Spain (Andalusia)

Víctor Jiménez-González [1,*], Guillermo Benítez [2], Julio Enrique Pastor [3], Miguel López-Lázaro [1] and José Manuel Calderón-Montaño [1,*]

[1] Department of Pharmacology, Faculty of Pharmacy, University of Seville, 41012 Seville, Spain; mlopezlazaro@us.es
[2] Department of Botany, Faculty of Pharmacy, University of Granada, 18071 Granada, Spain; gbcruz@ugr.es
[3] Department of Vegetal Biology and Ecology, Faculty of Biology, University of Seville, 41012 Seville, Spain; jpastor@us.es
* Correspondence: vjimenez3@us.es (V.J.-G.); jcalderon@us.es (J.M.C.-M.)

1. Supplementary Materials

Table S1. Geographical coordinates of the collection sites for the botanical species evaluated in this study.

Extract	Plant Name	Coordinates
**1-2**	*Acanthus mollis* L.	37°49'50.26"N 5°14'19.46"W
**3**	*Anemone palmata* L.	37°14'58"N 6°22'44"W
**4**	*Anogramma leptophylla* (L.) Link	37°24'09"N 6°01'45"W
**5**	*Antirrhinum cirrhigerum* (Welw. ex Ficalho) Rothm.	37º 23' 13"N 6º 0' 51" W
**6**	*Asphodelus fistulosus* L.	37º24'38"N 6º1'43"W
**7-8**	*Asphodelus ramosus* L.	37°49'39.34"N 5°14'19.68"W
**9**	*Ballota hirsuta* Benth.	37º 25' 22" N 5º 59' 47" W
**10**	*Bryonia cretica* subsp. *dioica* (Jacq.) Tutin	37º34'48"N 6º4'40"W
**11**	*Cardamine hirsuta* L.	37º24'1" N 6º14'2" W
**12-13**	*Carpobrotus edulis* (L.) N.E.Br.	37°17'47"N 6°23'26 W
**14**	*Cerinthe major* L.	37º 22' 33" N 6º 13' 7" W
**15**	*Clematis cirrhosa* L.	36º32'32"N 6º11'13"W
**16**	*Clematis flammula* L.	37°50'36.62"N 5°14'13.22"W
**17**	*Crambre filiformis* Jacq.	37º50'28" N 5º14'10" W
**18**	*Daphne gnidium* L.	37º30'54" N 6º23'33" W
**19-20**	*Delphinium pentagynum* Lam.	37°50'5.77"N 5°14'12.87"W
**21**	*Dioscorea communis* (L.) Caddick & Wilkin	37º36'15"N 6º3'11"W
**22**	*Dipcadi serotinum* (L.) Medik.	37°16'21"N 6°23'52"W
**23-24**	*Dolichandra unguis-cati* (L.) L.G.Lohmann*	37°21'46"N 5°59'14"W
**25**	*Foeniculum vulgare* Mill.	37º21'39"N 5º59'21" W
**26**	*Frankenia laevis* L.	36º31'14"N 6º12'22"W
**27**	*Geranium purpureum* Vill.	37º51'7" N 5º14'4" W
**28**	*Gladiolus × byzantinus* Mill.	37°39'28.9"N 6°13'37.2"W
**29**	*Globularia spinosa* L.	37°50'54.5"N 2°57'34.3"W
**30**	*Helianthemum angustatum* Pomel	37º21'10" N 5º47'51" W

Extract	Plant Name	Coordinates
**31**	*Helianthemum hirtum* (L.) Mill.	37º19'13'' N 5º46'20'' W
**32**	*Himantoglossum robertianum* (Loisel.) P.Delforge	37º35'49''N 6º2'59'' W
**33**	*Iberodes linifolia* (L.) M.Serrano, R.Carbajal & S.Ortiz	37º51'15''N 5º14'3'' W
**34**	*Chamaeris foetidissima* (L.) Medik. (=*Iris foetidissima* L.)	36°32'43"N 6°12'20"W
**35**	*Jasione montana* L.	37°49'50.26"N 5°14'19.46"W
**36**	*Lamarckia aurea* (L.) Moench	37°19'05.5"N 6°03'15.5"W
**37**	*Lepidium didymum* (L.) Sm.	37°25'48"N 5°57'44"W
**38**	*Linum appressum* Caball.	37°50'55.8"N 2°57'34.5"W
**39**	*Misopates calycinum* (Lange) Rothm.	37º39'30'' N 6º13'39'' W
**40-41**	*Nepeta tuberosa* L.	37º49'3'' N 5º14'21'' W
**42**	*Osyris alba* L.	37º50'56'' N 5º14'12'' W
**43**	*Parietaria hirsuta* L.	37º 21' 38'' N 5º59'18'' W
**44**	*Petrosedum forsterianum* (Sm.) Grulich	36º32'16''N 6º11'17''W
**45**	*Pteridium aquilinum* (L.) Kuhn	37º8'35''N 6º32'29''W
**46**	*Reseda media* Lag.	37º7'32''N 6º29'59''W
**47**	*Rumex bucephalophorus* L.	37°14'59"N 6°22'42"W
**48**	*Rumex spinosus* L.	37º21'39''N 5º59'21'' W
**49-50**	*Ruscus aculeatus* L.	37º8'35''N 6º32'29''W
**51**	*Ruta montana* (L.) L.	37º25'56''N 6º2'17'' W
**52**	*Saxifraga hirsuta* L.	37º36'21''N 6º3'29'' W
**53**	*Scilla peruviana* L.	36º32'14''N 6º12'22''W
**54**	*Selaginella denticulata* (L.) Spring	37º39'16''N 6º0'22''W
**55**	*Seseli montanum* subsp. *granatense* (Willk.) C. Pardo	37°50'56.2"N 2°57'35.5"W
**56**	*Smilax aspera* L.	37º8'35''N 6º32'29''W
**57**	*Thymelaea hirsuta* (L.) Endl.	36º 31' 56'' N 6º 12' 40'' W
**58**	*Tordylium officinale* L.	37º51'16''N 5º14'3'' W
**59**	*Trachelium caeruleum* L.	37º51'13'' N 5º14'2'' W
**60**	*Tradescantia pallida* (Rose) D.R.Hunt*	37º21'42'' N 5º 59' 10'' W
**61**	*Daphne oleoides* Schreb.	36°45'59.4"N 5°23'39.9"W
**62**	*Thymelaea elliptica* (Boiss.) Endl.	37º21'48'' N 2º53'31'' W
**63**	*Thymelaea granatensis* (Pau) Lacaita	37°50'55"N 2°57'35"W
**64**	*Thymelaea lanuginosa* (Lam.) Ceballos & C.Vicioso	37°14'30.4"N 5°59'47.5"W
**65**	*Thymelaea lythroides* Barratte & Murb.	37°16'13.4"N 5°27'37.6"W
**66-67**	*Thymelaea tartonraira* subsp. *austroiberica* Lambinon	37º5'7'' N 3º31'29'' W

(*) Non-native species of ornamental origin currently found in wild habitats.

Table S2. The extraction yields (%) obtained for each of the botanical extracts evaluated in the present study.

Extract	Plant Name	Part used	Extraction yield (%)
**1**	*Acanthus mollis* L.	Aerial part with flowers	3.8
**2**	*Acanthus mollis* L.	Fruits	8.7
**3**	*Anemone palmata* L.	Aerial part with flowers	4.8
**4**	*Anogramma leptophylla* (L.) Link	Aerial part	3.7
**5**	*Antirrhinum cirrhigerum* (Welw. ex Ficalho) Rothm.	Aerial plant with flowers	1.2
**6**	*Asphodelus fistulosus* L.	Aerial parts with flowers	6.2
**7**	*Asphodelus ramosus* L.	Leaves	2.2
**8**	*Asphodelus ramosus* L.	Flowers	1.9
**9**	*Ballota hirsuta* Benth.	Aerial part with flowers	4.0
**10**	*Bryonia cretica* subsp. *dioica* (Jacq.) Tutin	Aerial part	3.4
**11**	*Cardamine hirsuta* L.	Whole plant	3.7
**12**	*Carpobrotus edulis* (L.) N.E.Br.	Flowers	3.3
**13**	*Carpobrotus edulis* (L.) N.E.Br.	Leaves	4.0
**14**	*Cerinthe major* L.	Aerial parts with flowers	5.0
**15**	*Clematis cirrhosa* L.	Aerial part	1.3
**16**	*Clematis flammula* L.	Flowers	15.0
**17**	*Crambre filiformis* Jacq.	Aerial part with flowers	4.6
**18**	*Daphne gnidium* L.	Leaves	6.2
**19**	*Delphinium pentagynum* Lam.	Aerial part with flowers	6.2
**20**	*Delphinium pentagynum* Lam.	Flowers	6.9
**21**	*Dioscorea communis* (L.) Caddick & Wilkin	Aerial part	3.7
**22**	*Dipcadi serotinum* (L.) Medik.	Whole plant	10.4
**23**	*Dolichandra unguis-cati* (L.) L.G.Lohmann*	Flowers	6.6
**24**	*Dolichandra unguis-cati* (L.) L.G.Lohmann*	Leaves	3.9
**25**	*Foeniculum vulgare* Mill.	Aerial part	5.0
**26**	*Frankenia laevis* L.	Aerial part with flowers	8.7
**27**	*Geranium purpureum* Vill.	Aerial part	2.4
**28**	*Gladiolus × byzantinus* Mill.	Aerial parts with flowers	9.9
**29**	*Globularia spinosa* L.	Aerial part	8.7
**30**	*Helianthemum angustatum* Pomel	Aerial part with flowers	5.7
**31**	*Helianthemum hirtum* (L.) Mill.	Aerial part with flowers	4.7
**32**	*Himantoglossum robertianum* (Loisel.) P.Delforge	Aerial part with flowers	3.5
**33**	*Iberodes linifolia* (L.) M.Serrano, R.Carbajal & S.Ortiz	Aerial part with flowers	3.4
**34**	*Chamaeris foetidissima* (L.) Medik. (=*Iris foetidissima* L.)	Aerual part with fruits	3.2
**35**	*Jasione montana* L.	Aerial part with flowers	4.8
**36**	*Lamarckia aurea* (L.) Moench	Whole plant	5.8
**37**	*Lepidium didymum* (L.) Sm.	Whole plant	5.7
**38**	*Linum appressum* Caball.	Aerial part	3.6
**39**	*Misopates calycinum* (Lange) Rothm.	Whole plant	9.3
**40**	*Nepeta tuberosa* L.	Aerial part with flowers	6.0
**41**	*Nepeta tuberosa* L.	Roots	8.0

Extract	Plant Name	Part used	Extraction yield (%)
**42**	*Osyris alba* L.	Aerial part with flowers	16.6
**43**	*Parietaria hirsuta* L.	Whole plant	4.2
**44**	*Petrosedum forsterianum* (Sm.) Grulich	Whole plant	1.8
**45**	*Pteridium aquilinum* (L.) Kuhn	Aerial part	4.4
**46**	*Reseda media* Lag.	Whole plant	0.7
**47**	*Rumex bucephalophorus* L.	Aerial part with flowers	4.4
**48**	*Rumex spinosus* L.	Whole plant	4.2
**49**	*Ruscus aculeatus* L.	Leaves	0.3
**50**	*Ruscus aculeatus* L.	Fruits	4.6
**51**	*Ruta montana* (L.) L.	Aerial part with flowers	8.0
**52**	*Saxifraga hirsuta* L.	Whole plant	5.0
**53**	*Scilla peruviana* L.	Whole plant	6.5
**54**	*Selaginella denticulata* (L.) Spring	Whole plant	6.6
**55**	*Seseli montanum* subsp. *granatense* (Willk.) C. Pardo	Aerial part	7.8
**56**	*Smilax aspera* L.	Fruits	3.2
**57**	*Thymelaea hirsuta* (L.) Endl.	Aerial parts	2.9
**58**	*Tordylium officinale* L.	Aerial part with flowers and fruits	6.8
**59**	*Trachelium caeruleum* L.	Leaves	3.9
**60**	*Tradescantia pallida* (Rose) D.R.Hunt*	Aerial parts	2.1
**61**	*Daphne oleoides* Schreb.	Aerial part with flowers	6.3
**62**	*Thymelaea elliptica* (Boiss.) Endl.	Aerial part with flowers	2.7
**63**	*Thymelaea granatensis* (Pau) Lacaita	Aerial part	4.2
**64**	*Thymelaea lanuginosa* (Lam.) Ceballos & C.Vicioso	Aerial part	3.3
**65**	*Thymelaea lythroides* Barratte & Murb.	Aerial part	3.9
**66**	*Thymelaea tartonraira* subsp. *austroiberica* Lambinon	Aerial part	5.2
**67**	*Thymelaea tartonraira* subsp. *austroiberica* Lambinon	Bark	6.7

(*) Non-native species of ornamental origin currently found in wild habitats.

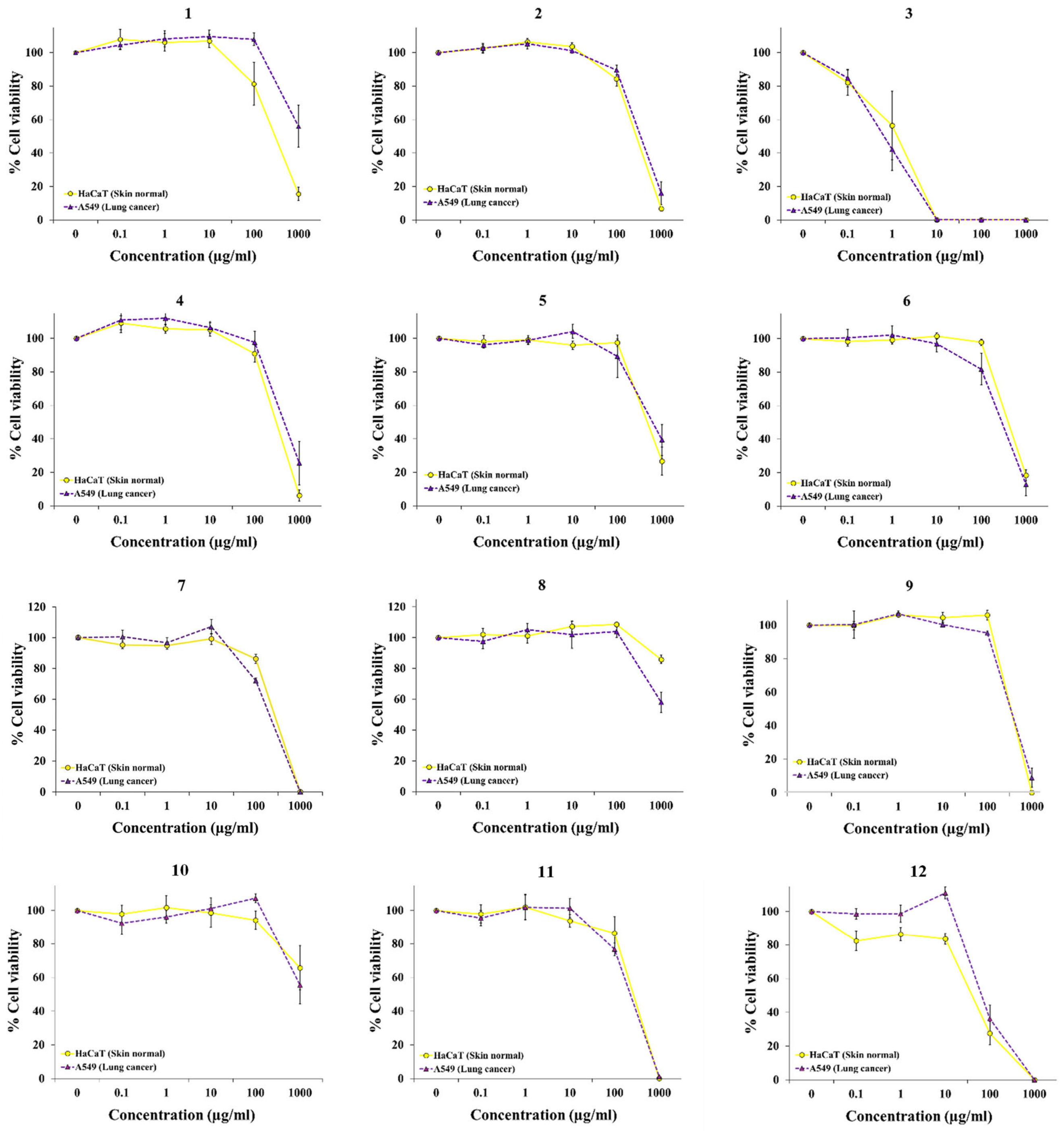

Figure S1. Assessment of the differential cytotoxic effects of extracts **1–12** on human lung adenocarcinoma (A549) and non-tumorigenic cells (HaCaT). Cell viability was performed after 72 h of treatment using the resazurin-based metabolic assay.

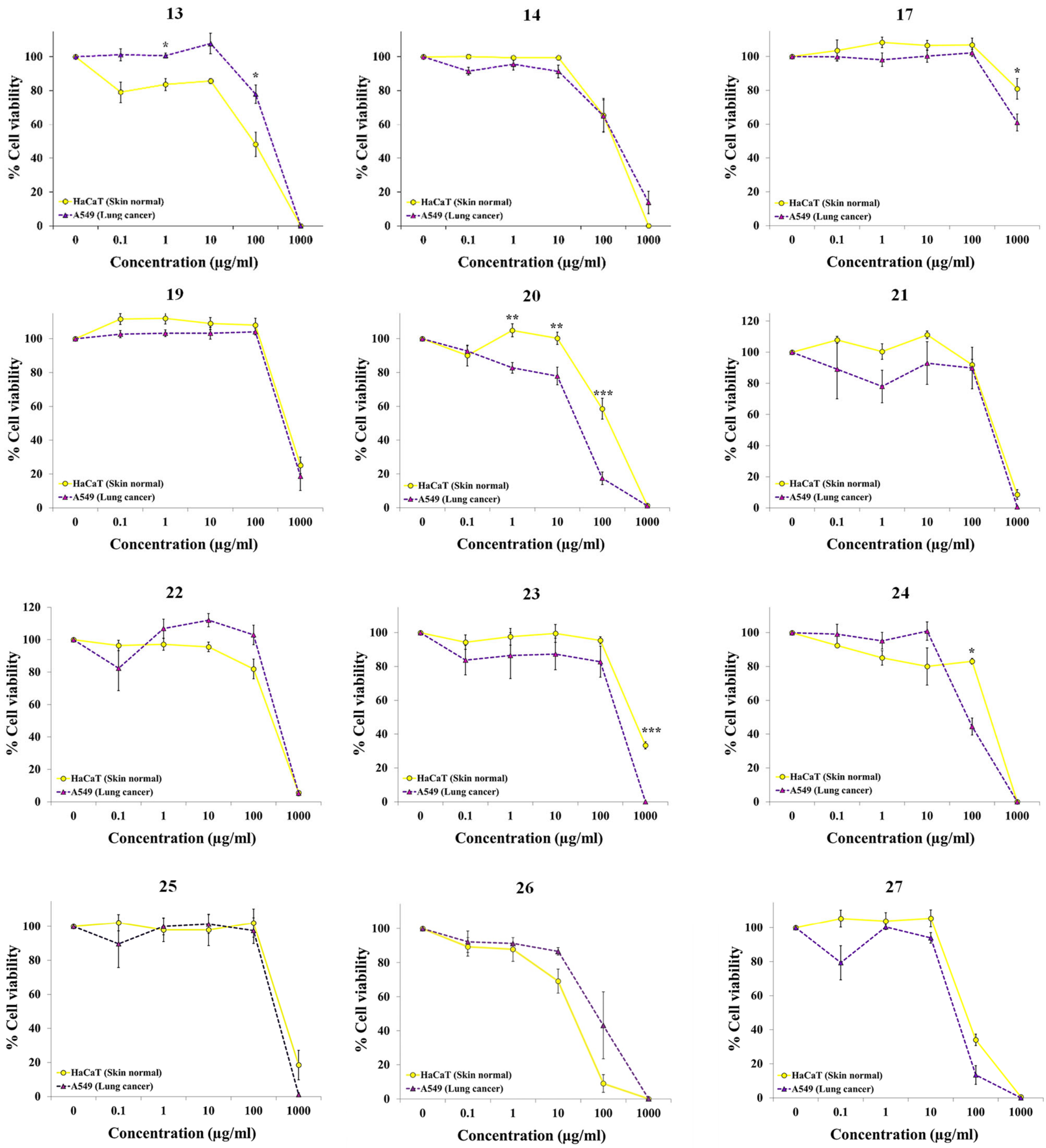

Figure S2. Assessment of the differential cytotoxic effects of extracts **13–14**, **17**, **19-27** on human lung adenocarcinoma (A549) and non-tumorigenic cells (HaCaT). Cell viability was performed after 72 h of treatment using the resazurin-based metabolic assay.

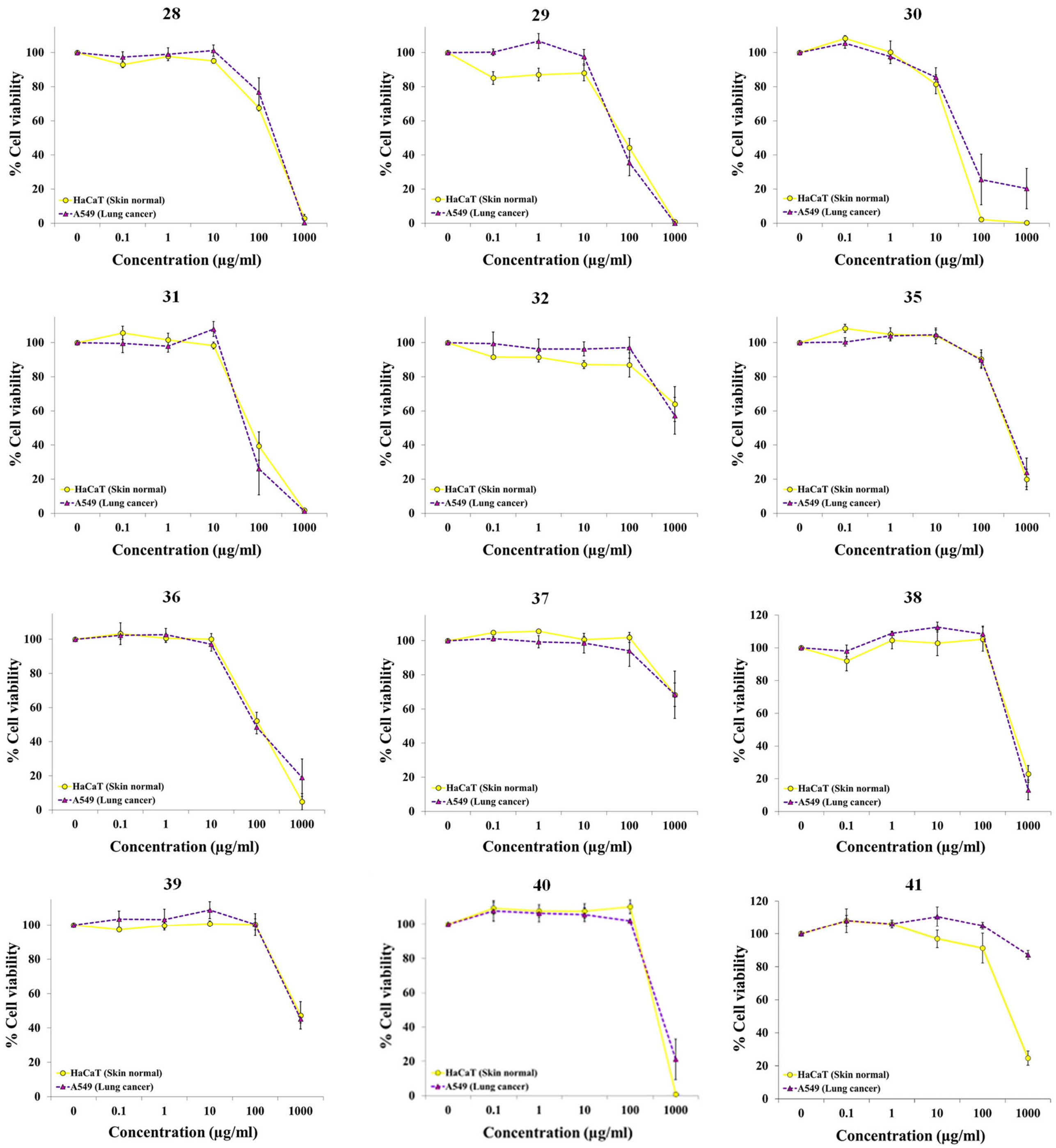

Figure S3. Assessment of the differential cytotoxic effects of extracts **28-32, 35-41** on human lung adenocarcinoma (A549) and non-tumorigenic cells (HaCaT). Cell viability was performed after 72 h of treatment using the resazurin-based metabolic assay.

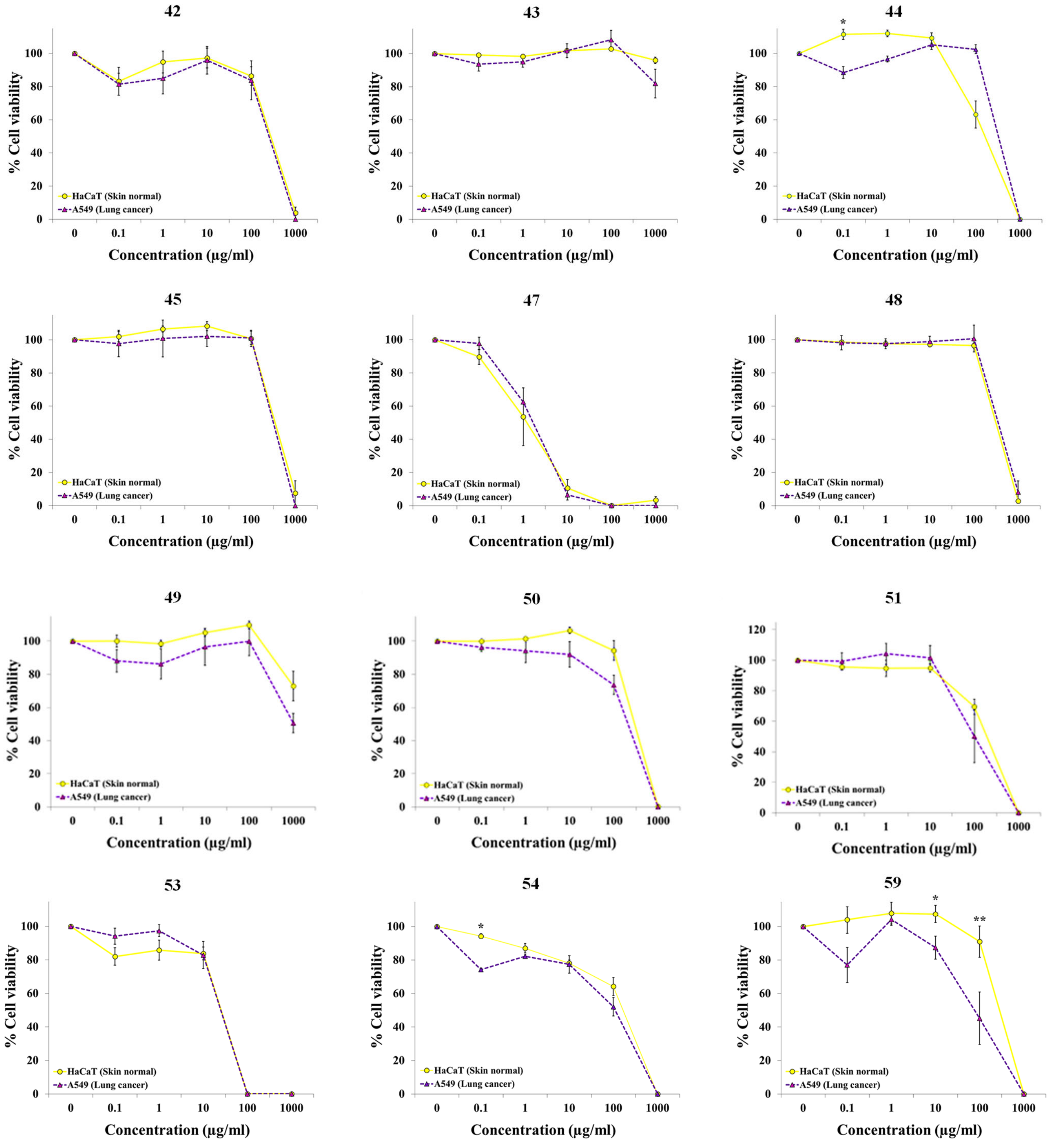

Figure S4. Assessment of the differential cytotoxic effects of extracts **42-45, 47-51, 53-54** and **59** on human lung adenocarcinoma (A549) and non-tumorigenic cells (HaCaT). Cell viability was performed after 72 h of treatment using the resazurin-based metabolic assay.

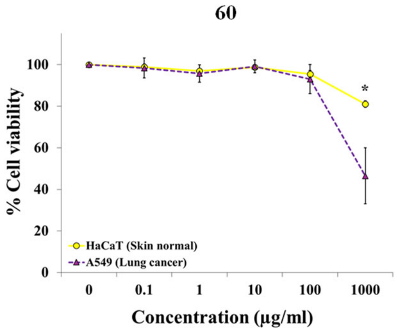

Figure S5. Assessment of the differential cytotoxic effects of extract **60** on human lung adenocarcinoma (A549) and non-tumorigenic cells (HaCaT). Cell viability was performed after 72 h of treatment using the resazurin-based metabolic assay.

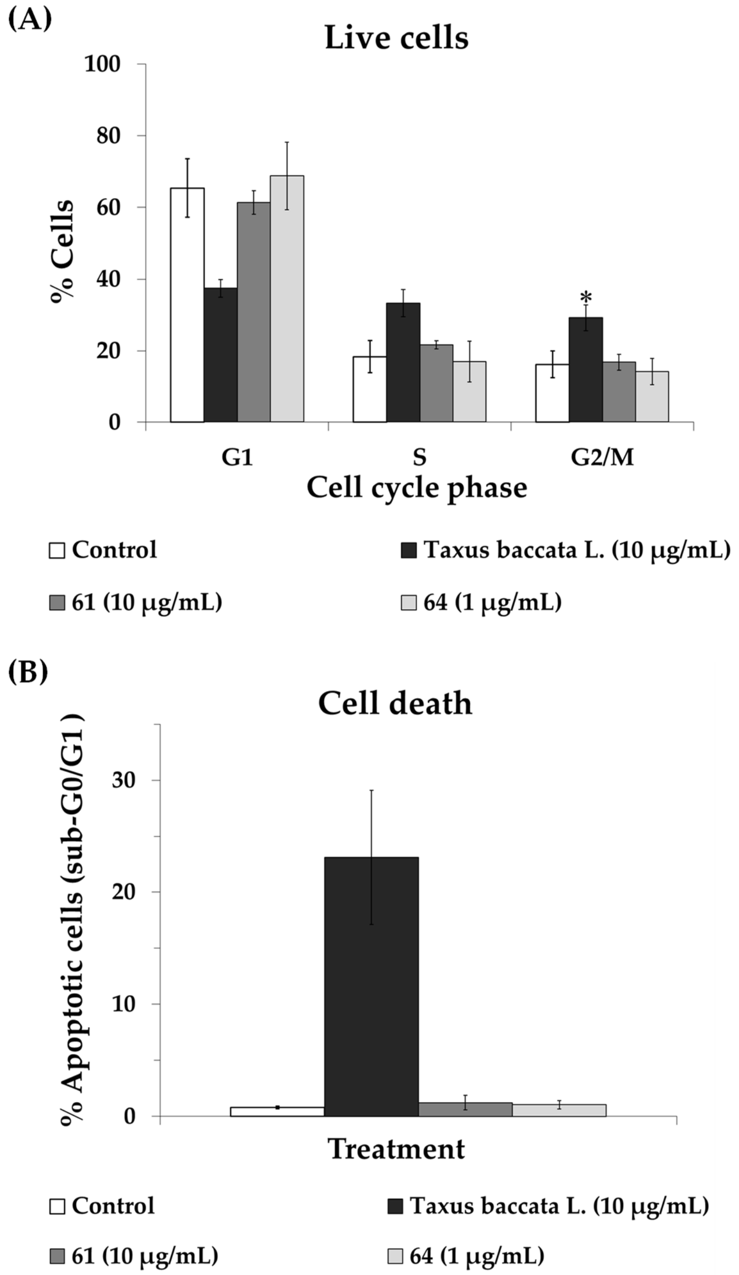

Figure S6. Effects of extract **61** (*Daphne oleoides*), **64** (*Thymelaea lanuginosa*) and *Taxus baccata* on cell cycle distribution in HaCaT cells following a 72-hour exposure. Post-treatment, DNA content was quantified via flow cytometry using propidium iodide staining. (**A**) Analysis of the cell-cycle profile in viable populations. (**B**) Quantification of the lethal fraction (dead cells), defined by a sub-G1 DNA content (<2N). Results are expressed as mean ± SEM of a minimum of two independent biological replicates. Statistical significance was determined by a paired t-test (* $p < 0.05$) compared to untreated cells.

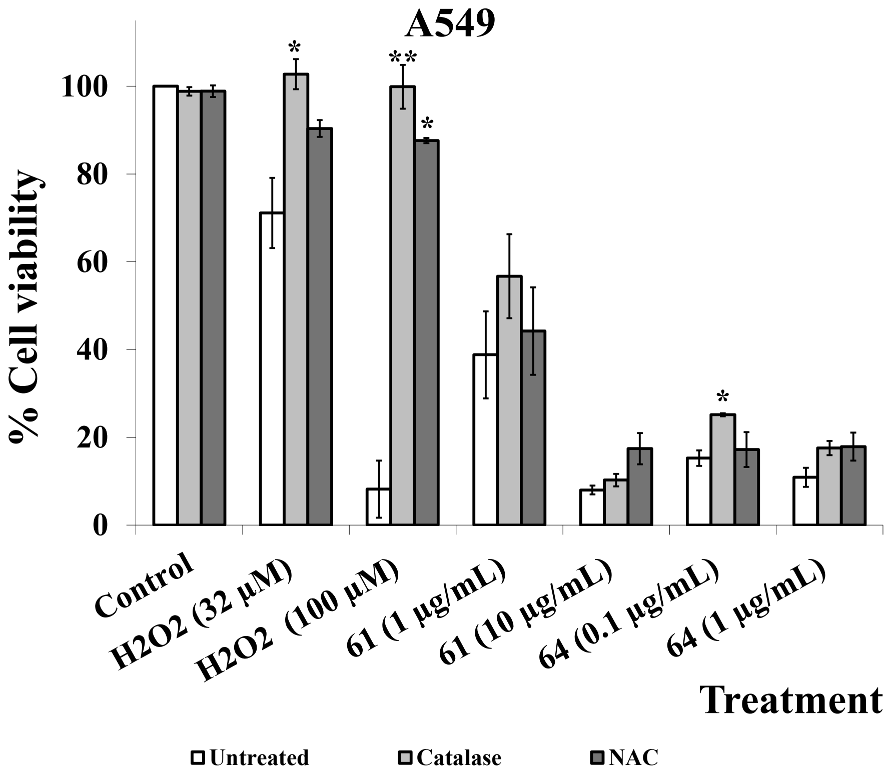

Figure S7. Assessment of the contribution of ROS generation to the cytotoxicity of the extract **61** (*Daphne oleoides*), **64** (*Thymelaea lanuginosa*) in A549 cells. Cells were treated with extracts or H_2O_2 for 72 hours in the absence or presence of catalase or N-acetylcysteine (NAC). Antioxidants were added 1 hour before extracts or H_2O_2. Results are expressed as mean ± SEM of a minimum of two independent biological replicates. Statistical significance was determined by a paired t-test (* $p < 0.05$; ** $p < 0.01$) compared to untreated cells (without antioxidant).
